# Timing and tempo of pubertal development and substance use in adolescence: a cohort study in the Danish National Birth Cohort

**DOI:** 10.1093/hropen/hoaf072

**Published:** 2025-11-18

**Authors:** Pernille Jul Clemmensen, Nis Brix, Anne Gaml-Sørensen, Anne Marie Ladehoff Thomsen, Thea Emily Benson, Andreas Ernst, Katrine Strandberg-Larsen, Cecilia Høst Ramlau-Hansen

**Affiliations:** Department of Public Health, Aarhus University, Aarhus C, Denmark; Department of Public Health, Aarhus University, Aarhus C, Denmark; Department of Clinical Genetics, Aarhus University Hospital, Aarhus N, Denmark; Department of Public Health, Aarhus University, Aarhus C, Denmark; Department of Public Health, Aarhus University, Aarhus C, Denmark; Department of Public Health, Aarhus University, Aarhus C, Denmark; Department of Public Health, Aarhus University, Aarhus C, Denmark; Department of Urology, Aarhus University Hospital, Aarhus N, Denmark; Section of Epidemiology, Department of Public Health, University of Copenhagen, Copenhagen K, Denmark; Department of Public Health, Aarhus University, Aarhus C, Denmark

**Keywords:** puberty, smoking, alcohol, drug use, risk-taking behaviours, cohort study, adolescence

## Abstract

**STUDY QUESTION:**

Are the age at reaching different pubertal milestones, and the tempo of pubertal progression, associated with cigarette smoking, alcohol intake, or recreational drug use in adolescence?

**SUMMARY ANSWER:**

Earlier age at pubertal development was associated with a higher risk of smoking cigarettes, drinking alcohol (only boys), and using recreational drugs in adolescence, and a faster tempo tended to be associated with higher risks, mainly in girls, while any associations in boys appeared less clear.

**WHAT IS KNOWN ALREADY:**

Girls and boys with an earlier age at pubertal development are suggested to have a higher risk of engaging in risk-taking behaviours, which include use of substances, primarily studied for smoking and alcohol. The potential impact of pubertal tempo has been studied less.

**STUDY DESIGN, SIZE, DURATION:**

In this cohort study, 8063 girls and boys born from 2000 to 2003 by mothers in the Danish National Birth Cohort participated. Information on covariates was obtained from pregnancy interviews and a follow-up questionnaire at age 7. Information on pubertal development was obtained from questionnaires completed by the girls and boys throughout puberty, starting at age 11, and information on substance use, covering cigarette smoking, alcohol, and recreational drug use, was obtained from a questionnaire completed at a mean age of 18 years and 4 months.

**PARTICIPANTS/MATERIALS, SETTING, METHODS:**

A multinomial logistic regression model was used to obtain adjusted relative risk ratios (RRRs) of smoking, drinking alcohol, and using recreational drugs (non-medical use of psychoactive substances) per 1 year earlier timing of pubertal development (Tanner stages of breast/genital and pubic hair development, menarche, first ejaculation, and voice break) and for tempo per one-unit (Tanner stages per year) faster tempo. Additionally, we also analysed categories of pubertal timing (15% earliest, 70% (ref.), 15% latest) and tempo (15% fastest, 70% (ref.), 15% slowest). The timing and tempo of the development of Tanner stages were derived using a non-linear mixed-effects model.

**MAIN RESULTS AND THE ROLE OF CHANCE:**

At the age of 18 years, 19% of girls and 20% of boys reported smoking at least monthly, 69% of girls and 71% of boys reported consuming alcohol more frequently than once a month, and 17% of girls and 30% of boys reported use of recreational drugs (primarily hash or pot) at some point during the past year. In girls, the RRR per 1-year earlier breast development was 1.20 (95% CI, 1.08–1.34) for smoking daily or weekly and 1.10 (95% CI, 0.97–1.25) for smoking monthly, and in boys, the RRR per 1-year earlier genital development was 1.09 (95% CI, 0.94–1.27) for smoking daily or weekly and 1.17 (95% CI, 1.02–1.34) for smoking monthly compared to no smoking. In girls, earlier age at menarche, but no other milestone, was associated with lower risk of alcohol consumption at age 18, whereas in boys, earlier age at genital development was associated with higher risk of drinking alcohol weekly (RRR 1.13 (95% CI, 0.99–1.30) per 1-year earlier timing) and 2–4 times/month (RRR 1.07 (95% CI, 0.96–1.19) per 1-year earlier timing) compared to drinking alcohol ≤1 time/month. In girls, the RRR per 1-year earlier breast development was 1.31 (95% CI, 1.07–1.59) for using recreational drugs monthly and 1.04 (95% CI, 0.93–1.15) for using recreational drugs <1 time/month. In boys, the RRR per 1-year earlier genital development was 1.17 (95% CI, 0.97–1.43) for using recreational drugs monthly and 1.16 (95% CI, 1.03–1.30) for using recreational drugs <1 time/month compared to no recreational drug use. Faster pubertal tempo tended to be associated with higher risk of smoking, drinking alcohol, and using recreational drugs, mainly in girls.

**LIMITATIONS, REASONS FOR CAUTION:**

Information on pubertal development was self-reported, introducing a risk of misclassification of pubertal timing and tempo. Furthermore, the self-reported information on smoking habits, alcohol intake, and recreational drug use might be under-reported due to social stigmatization. We were not able to explore associations with snus or e-cigarettes, although this may be highly relevant.

**WIDER IMPLICATIONS OF THE FINDINGS:**

The findings suggest that girls and boys with earlier and maybe faster pubertal development may be high-risk groups for smoking cigarettes, drinking alcohol, and using recreational drugs. The associations were observed across various pubertal milestones, and the consistent findings across these strengthen the conclusion and support what has previously been shown primarily for pubertal timing and smoking. Our findings on pubertal tempo suggest that preventive strategies could benefit from targeting early developers and also fast-developing girls. This study was performed in a Danish cohort, and the findings are likely transferable to other Western populations with a comparable smoking, alcohol, and drug use culture.

**STUDY FUNDING/COMPETING INTEREST(S):**

This study has been supported by the Lundbeck Foundation (R396-2022-265). The project was further co-funded by the European Union (ERC, BIOSFER, 101071773). Views and opinions expressed are those of the author(s) only and do not necessarily reflect those of the European Union or the European Research Council. Neither the European Union nor the granting authority can be held responsible for them. The authors have no conflicts of interest to declare.

**TRIAL REGISTRATION NUMBER:**

N/A.

WHAT DOES THIS MEAN FOR PATIENTS? Puberty is a crucial life phase for reproductive health. The ability to conceive or impregnate a woman develops, accompanied by significant changes to the physical appearance, as well as behavioural and psychological changes. It has been suggested that the age at which pubertal development occurs influences mental health and risk-taking behaviours in adolescence. Few studies have looked at both the timing and pace of puberty using multiple markers of pubertal development. To address this gap, we used data from 8063 girls and boys in the Danish National Birth Cohort, who provided information on their pubertal development throughout puberty and on substance use at age 18 years. This detailed information enabled us to investigate how both the timing and pace of pubertal development relate to substance use in adolescence. In this study, we found that girls and boys with a younger age of pubertal development more often reported smoking, alcohol use (among boys), and recreational drug use during adolescence compared to girls and boys developing at a later age. We also observed that especially girls who went through puberty faster tended to report these patterns more frequently. The findings from this study indicate that girls and boys experiencing earlier puberty could be a target group for preventive strategies regarding smoking initiation, alcohol consumption, and recreational drug use.

## Introduction

Substance use, such as cigarette smoking, alcohol consumption, and recreational drug use, represents a major health risk, and initiation at an early age has been linked with a higher risk of dependence in adulthood (Khuder *et al.*, 1999; Blomeyer *et al.*, 2013; Hall *et al.*, 2016; Mittal *et al.*, 2024). Therefore, identifying risk factors of early initiation is of significant public health interest. Timing of pubertal development, i.e. the age at which different pubertal milestones are reached, varies considerably among individuals, with timing of Tanner Stage 3 occurring across a span of ∼5 years (Parent *et al.*, 2003; Lunddorf *et al.*, 2024).

Puberty is a life phase with marked neurological, psychological, and physical changes (Grumbach, 2002), and the timing of these changes might impact future health and risk-taking behaviours (Golub *et al.*, 2008; Graber, 2013). Associations with risk-taking behaviour may vary depending on the milestones used to assess the developmental stage, since different hormonal pathways are involved (Wood *et al.*, 2019). Additionally, some milestones are externally visible, potentially influencing how the child is perceived and treated by their social environment, thereby potentially affecting the risk-taking behaviour. Previous epidemiological studies have primarily focused on a single measure of pubertal development and have found that earlier timing of puberty was associated with higher adolescent substance use, with smoking and alcohol being the most extensively studied (Wilson *et al.*, 1994; Bratberg *et al.*, 2007; Kaltiala-Heino *et al.*, 2011; Cance *et al.*, 2013; Kong *et al.*, 2013; Dudovitz *et al.*, 2015; Kim *et al.*, 2017; Silva-Gallardo and Maggs, 2023). These findings are particularly relevant for further exploration, given the declining age of pubertal onset in Western countries (Ong *et al.*, 2006; Euling *et al.*, 2008; Eckert-Lind *et al.*, 2020). Investigating the association with various pubertal milestones in the same cohort makes it possible to separate their potentially different effects on risk-taking behaviour.

Similar to the timing of puberty, the tempo of puberty, i.e. the pace of the developmental progression, which can be measured in Tanner stages per year, may influence risk-taking behaviour in adolescence (Marceau *et al.*, 2011). Previously performed studies have found that a faster tempo of puberty was associated with a lower level of psychosocial well-being in boys (Cheng *et al.*, 2020), and in girls, with an earlier age at moderate alcohol consumption (Wilson *et al.*, 1994); however, research on the tempo of puberty and substance use is limited.

Using prospectively collected data on pubertal development of girls and boys born into the Danish National Birth Cohort (DNBC), this study aimed to investigate whether timing and tempo of puberty were associated with (i) smoking cigarettes, alcohol consumption, and recreational drug use at age 18 years, (ii) ever having tried smoking or using recreational drugs, and (iii) the age of smoking and alcohol consumption debut.

## Materials and methods

This study was carried out in accordance with the principles of the Declaration of Helsinki. The data collection in the DNBC was approved by the Danish Data Protection Agency and the Committee on Health Research Ethics (KF 01-471/94). The DNBC participants provided informed consent at enrolment. This study was registered by the Danish Data Protection Agency at Aarhus University (Rec. no. 1150) and approved by the Steering Committee of the DNBC (Ref. no. 2023-03 and 2023-07).

### Study design and population

**Figure 1.**
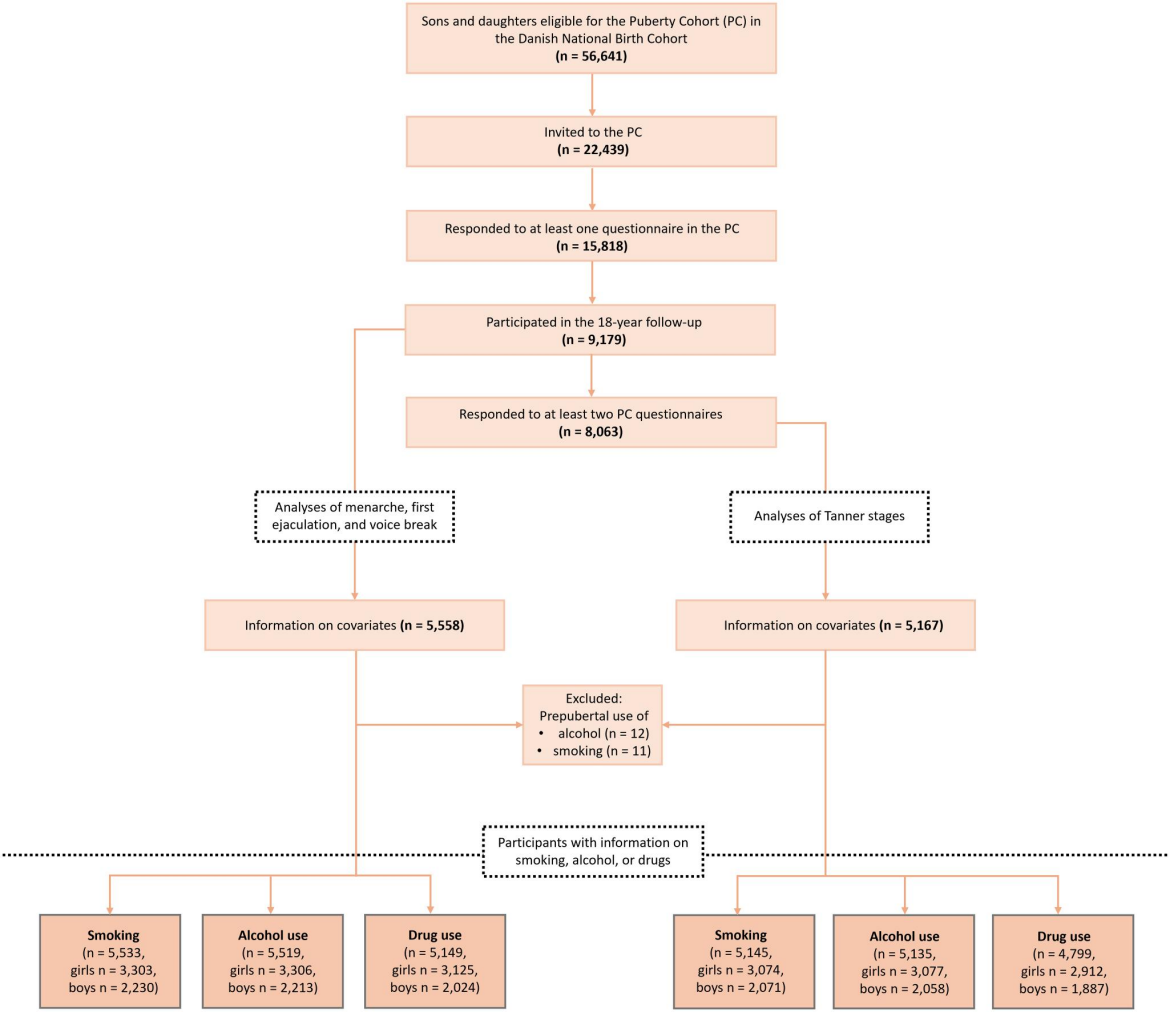
Flowchart of eligible study participants for studies on cigarette smoking, alcohol intake, and recreational drug use among young girls and boys participating in the Danish National Birth Cohort (DNBC) Puberty Cohort and the DNBC-18 questionnaire.

This population-based cohort study uses data from the DNBC. Approximately 30% of Danish pregnant women from 1996 to 2002 were enrolled (Olsen *et al.*, 2001), and their daughters and sons responded to several follow-up questionnaires. Among others, they responded to puberty questionnaires through puberty, including questions on attainment of Tanner stages for breast, genital, and pubic hair development and age at menarche, first ejaculation, and voice break. Whereas breasts, genitals, and pubic hair develop gradually over time, menarche, first ejaculation, and voice break are one-time events. Therefore, the analytical strategy, and consequently the study population, differs between analyses based on Tanner stage-defined milestones and those focusing on menarche, first ejaculation, and voice break. All participants in models with Tanner stages are also included in models with menarche, first ejaculation, and voice break, as illustrated in Fig. 1. To be included in the analyses of Tanner stages, participants had to have completed at least two questionnaires (n = 13 208) in the sub-cohort entitled the Puberty Cohort (Ernst *et al.*, 2020) and responded to the 18-year follow-up questionnaire (DNBC-18) sent out to the entire DNBC. A total of 8063 participants fulfilled these two criteria. Analyses of menarche, first ejaculation, and voice break included participants who responded to a single questionnaire in the Puberty Cohort (n = 15 818) together with the DNBC-18 questionnaire (n = 9179) (Fig. 1). Furthermore, participants had to have information on covariates from a pregnancy questionnaire completed around gestational Week 16 and a childhood questionnaire completed by the parents when the child turned 7 years (n = 5167 (Tanner stages)/5558 (other milestones)), along with information on the outcome: cigarette smoking (n = 5145/5533), alcohol intake (n = 5135/5519), and recreational drug use (n = 4799/5149). To limit the risk of reverse causation, participants who reported smoking their first cigarette before the age of 10 for girls and 11 for boys were excluded from the smoking analyses (n = 11), and participants reporting an intake of alcohol exceeding one unit before the same age were excluded from the alcohol analyses (n = 12). For drug use, information on debut age was not available. The cut-off values for age at smoking and alcohol use were selected based on mean ages at the timing of Tanner Stages B2 and G2 observed in the Puberty Cohort (Brix *et al.*, 2019a).The selection of the final study populations is illustrated in Fig. 1, including the distribution of sex for each outcome.

### Timing and tempo of pubertal development

Information on pubertal development in the children enrolled in the DNBC was collected for a subset of children born from 2000 to 2003. The children were invited to respond to a web-based questionnaire every 6 months about their current pubertal stages. The questionnaires were sent out for the first time when the child turned 11 years of age and ended when the child reported the completion of puberty (Tanner Stage 5 for pubic hair development and breast or genital development) or had turned 18 years of age. Information was collected on genital development (boys), voice break (boys), first ejaculation (boys), breast development (girls), menarche (girls), and pubic hair (both sexes). Questions on breast, genital, and pubic hair development were supported by illustrations of the different Tanner stages and reported as these: B1–5 (breast), G1–5 (genital), and PH1–5 (pubic hair) (Ernst *et al.*, 2020).

### Cigarette smoking, alcohol intake, and recreational drug use in adolescence

Information on cigarette smoking, alcohol intake, and recreational drug use was obtained from the DNBC-18 questionnaire, with the participants having a mean age of 18 years and 4 months (SD, 2 months) at the time of response. Because some response categories contained few participants, we combined certain categories as described below. Participants provided information on whether they had ever smoked a whole cigarette, and, if affirmative, the age in years at which they first did so, as well as the frequency of current cigarette smoking with the following response categories: (1) Daily, (2) Weekly, (3) Monthly, and (4) No current smoking. For the analyses, we combined Groups 1 and 2 into a single category.

Regarding alcohol intake, participants were asked whether they had ever consumed more than one unit on a single occasion, and, if affirmative, the age in years at which they first did so, as well as the frequency of current alcohol intake, based on the intake during the past year, with the following response categories: (1) Never, (2) No more than once a month, (3) 2–4 times per month, (4) 2–3 times per week, and (5) 4 or more times per week. For the analyses, we combined Groups 1 and 2 into a single category and Groups 4 and 5 into another category.

Recreational drug use was assessed by asking the participants if they had ever tried one or more of the following drugs: *hash or pot, amphetamines (speed), ecstasy/MDA, GHB (fantasy), sedatives or sleeping pills, cocaine, LSD, opiates, psychedelic mushrooms, inhalation of solvents or lighter gas, or inhalation of nitrous oxide*. Participants reporting recreational drug use were asked to report their use during the past year with the following response categories: (1) Not within the past year, (2) Only once, (3) Less than once a month, (4) Monthly, (5) Weekly, and (6) Daily or almost daily. For the analyses, we combined Groups 2 and 3 into a single category and Groups 4–6 into another category. Among participants with current drug use, 76% only used hash or pot, 5% only used one or more other drugs, while 19% used both hash or pot and one or more other drugs. Due to the small number of participants using other drugs, we grouped all drugs into a single outcome; therefore, we performed an additional analysis including only hash and pot use.

### Covariates

A directed acyclic graph illustrates the potential causal structures between timing and tempo of puberty and smoking cigarettes, drinking alcohol, and using recreational drugs in adolescence (Supplementary Fig. S1) (Greenland *et al.*, 1999). Based on this, the following covariates were chosen as potential confounding variables: first-trimester maternal smoking and alcohol intake, the highest educational level of the parents (data from the first DNBC pregnancy questionnaire), cohabitation of parents, maternal smoking, paternal smoking, the child’s externalizing and internalizing symptoms based on the Strengths and Difficulties Questionnaire (Goodman, 1997), maternal alcohol intake, and paternal alcohol intake (data from the DNBC 7-year questionnaire).

### Statistics

#### Estimation of mean age and tempo at mid-puberty for Tanner stages

As previous research has described pubertal development as a non-linear process, we used a non-linear mixed-effects model to obtain individual S-shaped trajectories for the longitudinal development of genitals (boys), breasts (girls), and pubic hair (girls and boys separately) from Tanner Stage 1 (pre-pubertal stage) to Tanner Stage 5 (fully matured stage) (Marceau *et al.*, 2011) based on the available data points. To increase individual-level precision, only participants with information on pubertal development from at least two puberty questionnaires were included in the model (n = 8063). The non-linear mixed-effects model is based on two parameters—the mean age at puberty (i.e. at Tanner Stage 3) and the tempo (i.e. the pace of progression through puberty, expressed as the slope at Tanner Stage 3). Each parameter has a fixed effect, which estimates the population mean of timing and tempo, and a normally distributed random effect, which estimates the individual deviation from the population mean of timing and tempo. The two random effects were allowed to covary. To obtain individual Tanner trajectories in terms of the timing and tempo, the Lindstrom–Bates algorithm was used, which is essentially the same as the Best Linear Unobserved Prediction used in linear mixed-effects models (Marceau *et al.*, 2011; Lunddorf *et al.*, 2024). Subsequently, timing of and tempo at mid-puberty were modelled as the exposure both continuously and additionally categorized into three categories each: 15% with the earliest timing (mean age: 10.6 for girls (Tanner stage B3), 11.4 for boys (Tanner stage G3)), 70% with average timing (mean age: 12.3 for girls (Tanner stage B3), 13.0 for boys (Tanner stage G3)), and 15% with the latest timing (mean age: 14.2 for girls (Tanner stage B3), and 14.9 for boys (Tanner stage G3)). A similar approach was used for tempo: 15% with the fastest tempo, 70% with average tempo, and 15% with the slowest tempo. For each category, the ranges, mean, and SD are provided in Supplementary Table S1. The categorization was based on the distribution of the DNBC mothers’ self-reported age at menarche, classified as earlier than, the same as, or later than peers (Sørensen *et al.*, 2018).

#### Imputation of age at menarche, first ejaculation, and voice break

The data collection structure of pubertal milestones resulted in left-, right-, and interval-censored data (Ernst *et al.*, 2020). If a participant had already obtained a milestone by the first puberty questionnaire, the age at that first response served as the upper bound, and the milestone was treated as left-censored (0% of girls for menarche and 0.5% of boys for first ejaculation). When the milestone occurred between two questionnaire responses, it was treated as interval-censored (3% of girls for menarche and 2% of boys for first ejaculation). If the milestone had not occurred by the final questionnaire, the age at the last response was used as the lower bound, and the milestone was treated as right-censored (20% of girls for menarche and 37% of boys for first ejaculation). The remaining participants, 77% of girls and 61% of boys, reported the exact age at menarche or first ejaculation. For voice break, only lower and/or upper age boundaries were available, with no exact ages reported. Therefore, we used multiple imputation by chained equations (imputation of 100 datasets) to estimate a specific age for individuals without an exact age reported. The imputation model incorporated the observed lower and upper limits of the pubertal milestones as boundaries by applying interval-censored regression. Covariates used for the imputation model are provided in Supplementary Table S2.

#### Inverse probability weights

Participants in the Puberty Cohort were sampled for invitation based on their exposure status to predefined exposures of interest. To account for this, each participant was assigned a sampling weight based on their probability of being invited to the Puberty Cohort (Brix *et al.*, 2019b). To further address potential selection bias due to nonparticipation, we estimated and applied selection weights (Hernan *et al.*, 2004). A description of the calculation of these is provided in Supplementary File S1.

#### Main analysis

We investigated whether the timing of Tanner Stages B3, G3, and PH3, and age at menarche, first ejaculation, and voice break were associated with smoking cigarettes, drinking alcohol, and using recreational drugs at the age of 18 years. A multinomial logistic regression model was used to obtain adjusted relative risk ratios (RRRs) with 95% CI per year earlier timing of the milestones for smoking cigarettes ‘weekly or daily’ or ‘monthly’ compared to not smoking, drinking alcohol ‘weekly’ or ‘2–4 times/month’ compared to drinking alcohol ≤1 time/month, where the latter also included those with no intake, and using drugs ‘monthly’ or ‘<1 time/month’ compared to no drug use.

Similarly, for tempo (Tanner stages per year), RRR of smoking was obtained per one-unit faster tempo of breast, genital, and pubic hair development at mid-puberty. In addition to the continuous models, timing and tempo of puberty were categorized, and RRRs of smoking, drinking alcohol, or using recreational drugs were estimated for each of the predefined groups (earliest or fastest 15% and latest or slowest 15%) compared to the reference group, which had either an average timing or tempo (mid 70% of the distribution).

#### Sub-analyses

In the first sub-analysis, we investigated whether the participants had ever tried smoking a whole cigarette or used any recreational drugs. Since almost all participants had tried to drink alcohol, we did not investigate whether they had ever tried to drink alcohol. For each outcome separately, a binomial regression model was used to obtain adjusted risk ratios (RRs) of the association between having tried vs not having tried per year earlier pubertal timing and per one unit faster pubertal development. Additionally, the RRs for each pubertal developmental group (relative to average timing and tempo) were calculated.

In the second sub-analysis, age at smoking a whole cigarette or having an alcohol intake exceeding one unit was investigated in relation to pubertal timing and tempo. Recreational drug use was not investigated due to data limitations. Using a binomial regression model, adjusted RRs of smoking debut < age 16 were compared to debut ≥ age 16 among participants who had tried to smoke a whole cigarette. Among participants who had tried to have an alcohol intake exceeding one unit, adjusted RRs of debut < age 16 were compared to debut ≥ age 16. A cut-off of 16 years was chosen, since this is the legal age for purchasing alcohol in Denmark. Like the previous analyses, the association was examined using pubertal timing and tempo as continuous and categorical variables.

For some pubertal milestones, the statistical model only converged after modifications were made to the number and levels of covariate categories. These modifications are indicated in footnotes to tables and figures. To illustrate the impact of this approach, we tried to make different confounder adjustments for the association between genital timing and ever having tried to smoke. These modifications had only a very limited impact on the results (Supplementary Table S3).

## Results

At the age of 18 years, 19% of girls and 20% of boys reported smoking cigarettes at least once a month, while 47% of girls and 48% of boys had tried smoking a whole cigarette at some point. At the age of 18 years, 69% of girls and 71% of boys reported alcohol intake more than once a month during the past year, and 96% of girls and 95% of boys had tried to have an alcohol intake exceeding one unit at some point. At the age of 18 years, 17% of girls and 30% of boys reported that they had used recreational drugs at least once during the past year, while 25% of girls and 39% of the boys had tried it at some point.

Overall, girls and boys who experienced earlier age at pubertal timing had mothers with lower alcohol intake during childhood, parents who smoked more and were less likely to cohabit, and, among girls, parents with lower educational status. Girls and boys with an earlier age at pubertal development also reported higher externalizing symptom scores at age 7 (Table 1).

**Table 1.** Baseline characteristics of girls and boys responding to questionnaires in the Danish National Birth Cohort (DNBC) Puberty Cohort at least twice and the DNBC-18 questionnaire (N = 8063).

	Girls, n = 4935			Boys, n = 3128			Total
	Timing, Tanner stage B3 (mean age (years))			Timing, Tanner stage G3 (mean age (years))			n = 8063
	Earliest (10.6)	Average (12.3)	Latest (14.2)	Earliest (11.4)	Average (13.0)	Latest (14.9)	
n (%)	740 (15)	3455 (70)	740 (15)	469 (15)	2190 (70)	469 (15)	
							Missing, %^a^
**Characteristics, pregnancy**							
**Highest socioeconomic position of the parents, n (%)**							0%
High-grade professional	146 (19.7)	834 (24.1)	206 (27.8)	134 (28.6)	559 (25.5)	126 (26.9)	
Low-grade professional	254 (34.3)	1213 (35.1)	257 (34.7)	140 (29.9)	762 (34.8)	177 (37.7)	
Skilled worker	206 (27.8)	885 (25.6)	196 (26.5)	121 (25.8)	563 (25.7)	117 (24.9)	
Unskilled worker	115 (15.5)	420 (12.2)	63 (8.5)	61 (13.0)	248 (11.3)	34 (7.2)	
Student	19 (2.6)	103 (3.0)	18 (2.4)	13 (2.8)	58 (2.6)	15 (3.2)	
**Daily number of maternal cigarettes in first trimester, n (%)**							∼0%
Non-smoker	473 (75.4)	2505 (72.7)	588 (79.5)	317 (67.6)	1618 (74.2)	378 (80.6)	
0–10	200 (27.0)	749 (21.7)	128 (17.3)	121 (25.8)	462 (21.2)	77 (16.4)	
>10	67 (9.1)	192 (5.6)	24 (3.2)	31 (6.6)	102 (4.7)	14 (3.0)	
**Weekly number of maternal alcohol units in first trimester, n (%)**							∼0%
0	385 (52.0)	≤1784 (≤52)	388 (52.4)	227 (48.4)	≤1132 (≤52)	225 (48.0)	
1	234 (31.6)	≥1082 (≥31)	246 (33.2)	153 (32.6)	≥683 (≥31)	165 (35.2)	
2–3	81 (10.9)	425 (12.3)	79 (10.7)	58 (12.4)	251 (11.5)	52 (11.1)	
>3	40 (5.4)	164 (4.7)	27 (3.6)	31 (6.6)	124 (5.7)	27 (5.8)	
**Characteristics, 7-year follow-up**							
**Daily number of maternal cigarettes, n (%)**							∼23%
Non-smoker	421 (75.4)	2112 (82.2)	510 (87.9)	282 (76.8)	1439 (83.2)	322 (84.3)	
0–10	61 (10.9)	207 (8.1)	37 (6.4)	33 (9.0)	121 (7.0)	29 (7.6)	
>10	76 (13.6)	251 (9.8)	33 (5.7)	52 (14.2)	169 (9.8)	31 (8.1)	
**Daily number of paternal cigarettes, n (%)**							∼25%
Non-smoker	398 (73.4)	1931 (77.0)	488 (85.0)	258 (73.9)	1351 (79.8)	311 (82.3)	
0–10	34 (6.3)	180 (7.2)	28 (4.9)	24 (6.9)	94 (5.5)	22 (5.8)	
>10	110 (20.3)	397 (15.8)	58 (10.1)	67 (19.2)	249 (14.7)	45 (11.9)	
**Weekly number of maternal alcohol units, n (%)**							∼23%
0	238 (42.5)	993 (38.6)	207 (35.6)	139 (37.6)	654 (37.8)	135 (∼35)	
1–14	313 (55.9)	1549 (60.2)	364 (62.7)	226 (61.1)	1059 (61.2)	≥241 (≥63)	
>14	9 (1.6)	30 (1.2)	10 (1.7)	5 (1.4)	17 (1.0)	≤5 (≤1)	
**Weekly number of paternal alcohol units, n (%)**							∼27%
0	116 (22.7)	517 (21.4)	113 (20.0)	70 (20.3)	341 (20.6)	69 (19.1)	
1–14	353 (69.1)	1697 (70.1)	399 (70.7)	246 (71.5)	1189 (72.0)	263 (72.9)	
>14	42 (8.2)	207 (8.6)	52 (9.2)	28 (8.1)	122 (7.4)	29 (8.0)	
**Parental cohabitation, n (%)**							∼23%
No	98 (17.4)	391 (15.1)	62 (10.7)	57 (15.4)	204 (11.7)	44 (11.4)	
Yes	466 (82.6)	2201 (84.9)	519 (89.3)	313 (84.6)	1544 (88.3)	342 (88.6)	
**Externalizing symptom score, n (%)** ^b^							∼23%
Low score	491 (87.8)	2310 (90.3)	525 (90.8)	319 (86.4)	1532 (88.6)	346 (91.3)	
High score	68 (12.2)	248 (9.7)	53 (9.2)	50 (13.6)	197 (11.4)	33 (11.3)	
**Internalizing symptom score, n (%)** ^b^							∼23%
Low score	499 (89.3)	2298 (89.7)	524 (91.3)	322 (87.7)	1542 (89.1)	326 (86.0)	
High score	60 (10.7)	263 (10.3)	50 (8.7)	45 (12.3)	189 (10.9)	53 (14.0)	

a To meet the General Data Protection Regulation (GDPR, Regulation (EU), 2016/679 of 25 May 2018) columns with missing data below n = 5 have been modified by rounding numbers in two cells up and down.

b Externalizing and internalizing symptom scores are based on the Strengths and Difficulties Questionnaire. In this study, high internalizing scores include scores ≥6 and high externalizing scores for girls scores ≥7 and for boys ≥8.

### Smoking

**Figure 2.**
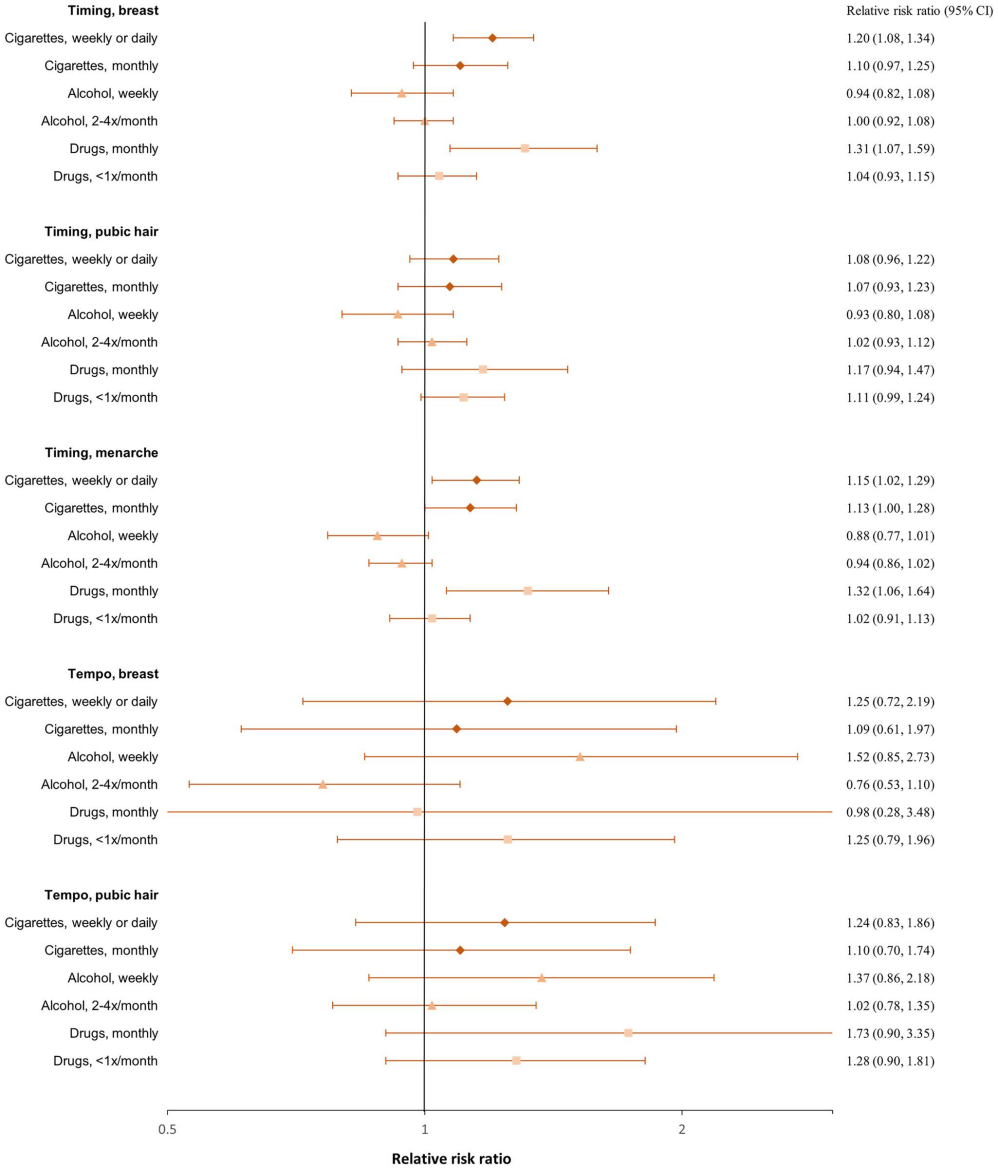
**Girls’ substance use at age 18**. Adjusted^a^ relative risk ratios with 95% confidence intervals for smoking cigarettes, drinking alcohol, or using drugs at age 18 compared to not smoking, not drinking alcohol (≤1 time/month), or not using drugs, across pubertal developmental groups. For timing, the estimates represent the relative risk ratios per year earlier pubertal development, and for tempo, per Tanner stages per year (indicating a faster progression). Estimates with 95% confidence intervals are provided in the right column. ^a^Adjusted for maternal alcohol intake in pregnancy, maternal smoking in pregnancy, parental socioeconomic status in pregnancy, parental cohabitation in childhood, childhood internalizing and externalizing symptoms and behaviours, paternal and maternal smoking in childhood, and paternal and maternal alcohol intake in childhood.

**Figure 3.**
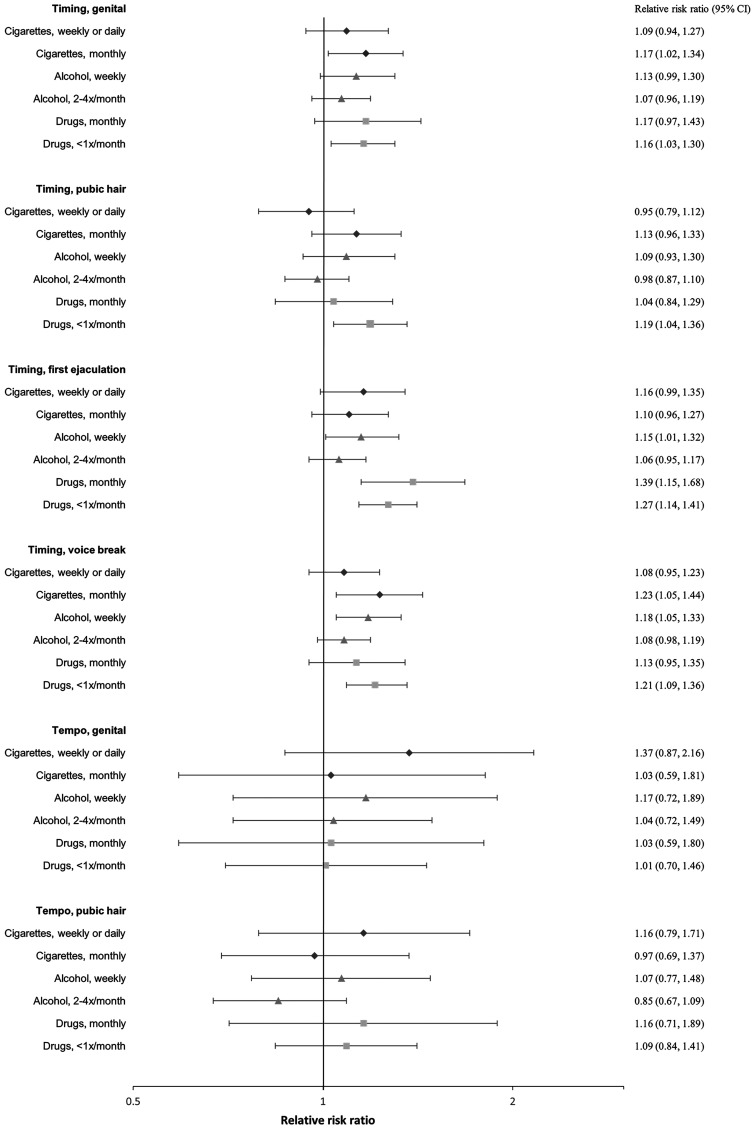
**Boys’ substance use at age 18**. Adjusted^a^ relative risk ratios with 95% confidence intervals for smoking cigarettes, drinking alcohol, or using drugs at age 18 compared to not smoking, not drinking alcohol (≤1 time/month), or not using drugs, across pubertal developmental groups. For timing, the estimates represent the relative risk ratios per year earlier pubertal development, and for tempo, per Tanner stages per year (indicating a faster progression). Estimates with 95% confidence intervals are provided in the right column. ^a^Adjusted for maternal alcohol intake in pregnancy, maternal smoking in pregnancy, parental socioeconomic status in pregnancy, parental cohabitation in childhood, childhood internalizing and externalizing symptoms and behaviours, paternal and maternal smoking in childhood, and paternal and maternal alcohol intake in childhood.

**Figure 4.**
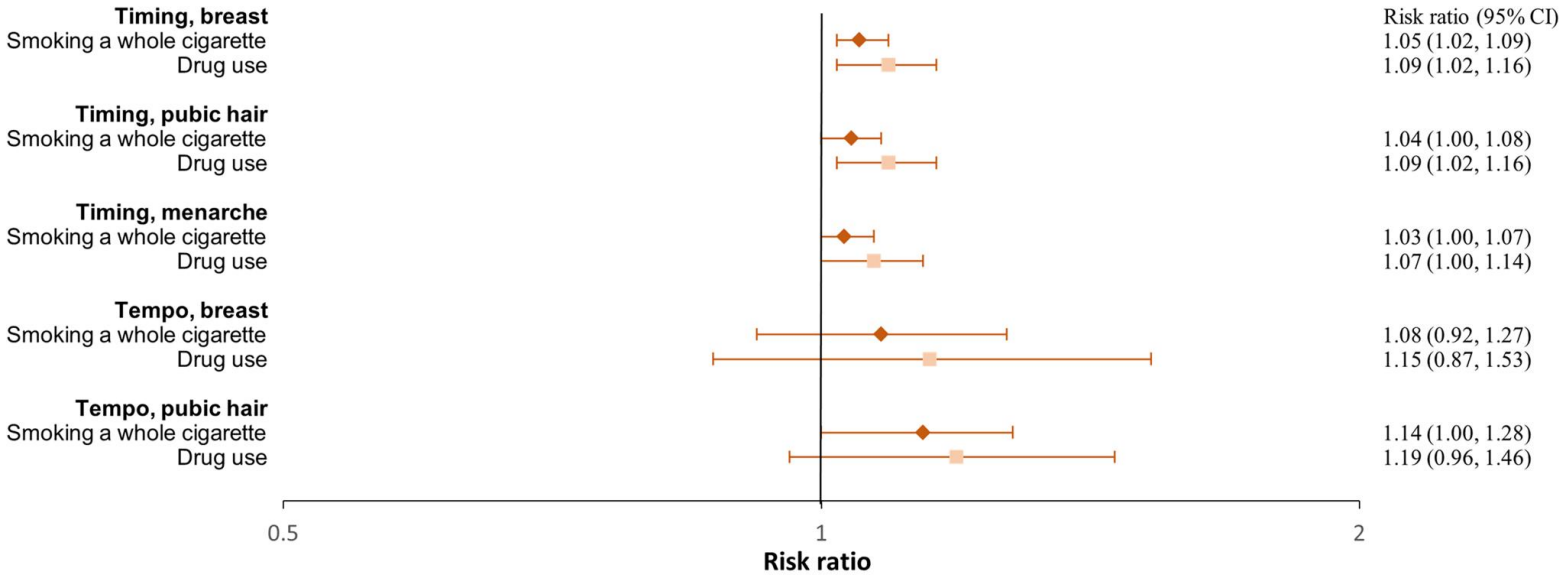
**Ever having used substances in girls**. Adjusted^a^ risk ratios with 95% confidence intervals of ever having tried smoking a whole cigarette or using drugs compared to not having tried across pubertal developmental groups. For timing, the estimates represent the risk ratios per year earlier pubertal development, and for tempo, per Tanner stages per year (indicating a faster progression). Estimates with 95% confidence intervals are provided in the right column. ^a^Adjusted for maternal alcohol intake in pregnancy, maternal smoking in pregnancy, parental socioeconomic status in pregnancy, parental cohabitation in childhood, childhood internalizing and externalizing symptoms and behaviours, paternal and maternal smoking in childhood, and paternal and maternal alcohol intake in childhood.

**Figure 5.**
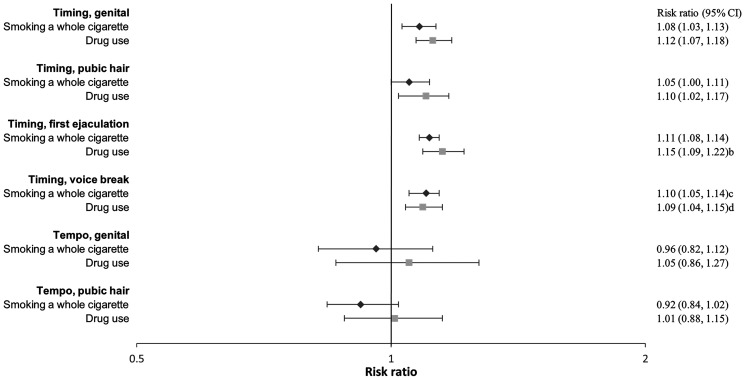
**Ever having used substances in boys**. Adjusted^a^ risk ratios with 95% confidence intervals of ever having tried smoking a whole cigarette or using drugs compared to not having tried across pubertal developmental groups. For timing, the estimates represent the risk ratios per year earlier pubertal development, and for tempo, per Tanner stages per year (indicating a faster progression). Estimates with 95% confidence intervals are provided in the right column. ^a^Adjusted for maternal alcohol intake in pregnancy, maternal smoking in pregnancy, parental socioeconomic status in pregnancy, parental cohabitation in childhood, childhood internalizing and externalizing symptoms and behaviours, paternal and maternal smoking in childhood, and paternal and maternal alcohol intake in childhood. ^b^Not adjusted for parental alcohol intake in childhood. ^c^Parental smoking in childhood (yes/no) and alcohol intake in childhood (yes/no) were dichotomised. Not adjusted for cohabitation and internalizing symptoms in childhood. ^d^Not adjusted for parental smoking and alcohol intake in childhood.

**Figure 6.**
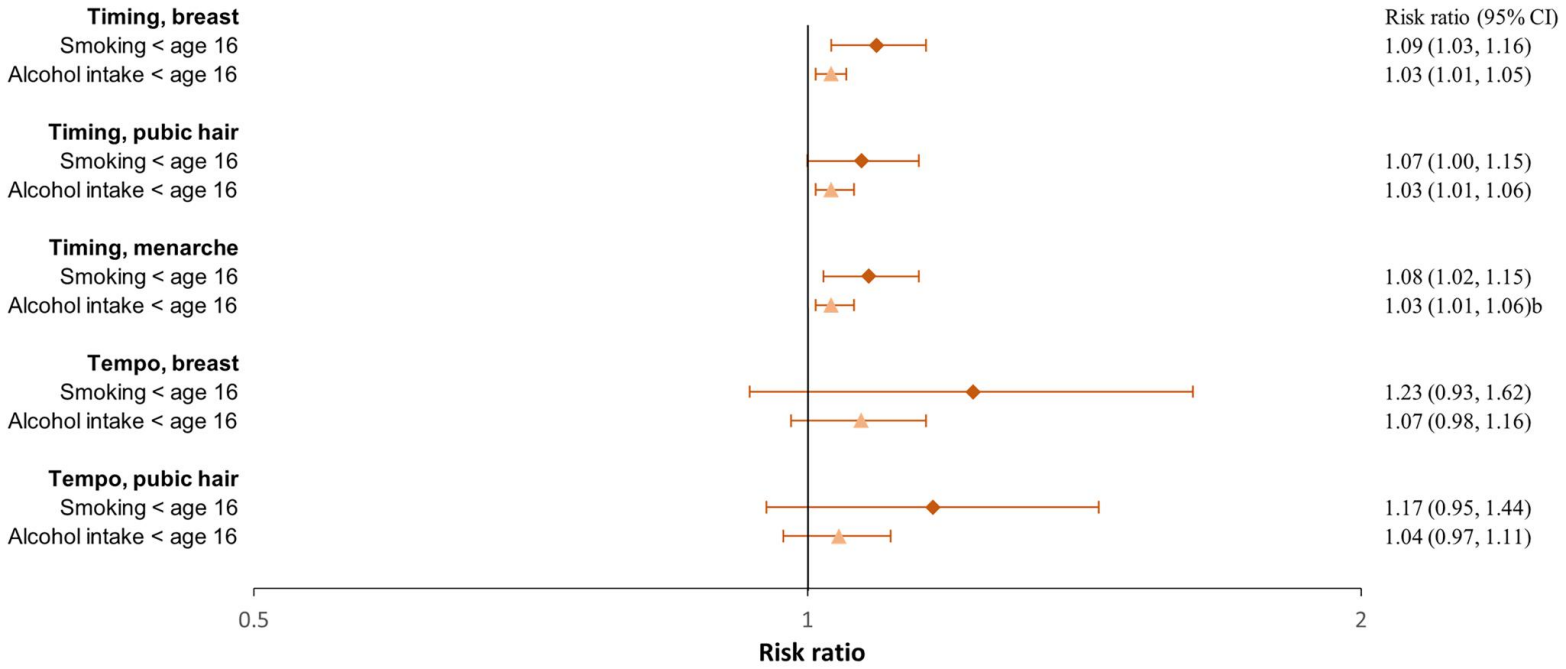
**Early debut of substance use in girls**. Age at smoking a whole cigarette or having an alcohol intake exceeding one unit across pubertal developmental groups. Adjusted^a^ risk ratios with 95% confidence interval of debut < age 16 compared to debut ≥ age 16. The estimates represent the risk ratios per year earlier pubertal development. Estimates with 95% confidence intervals are provided in the right column. ^a^Adjusted for maternal alcohol intake in pregnancy, maternal smoking in pregnancy, parental socioeconomic status in pregnancy, parental cohabitation in childhood, childhood internalizing and externalizing symptoms and behaviours, parental smoking in childhood (yes/no), and parental alcohol intake in childhood (yes/no). ^b^Not adjusted for parental smoking in childhood.

**Figure 7.**
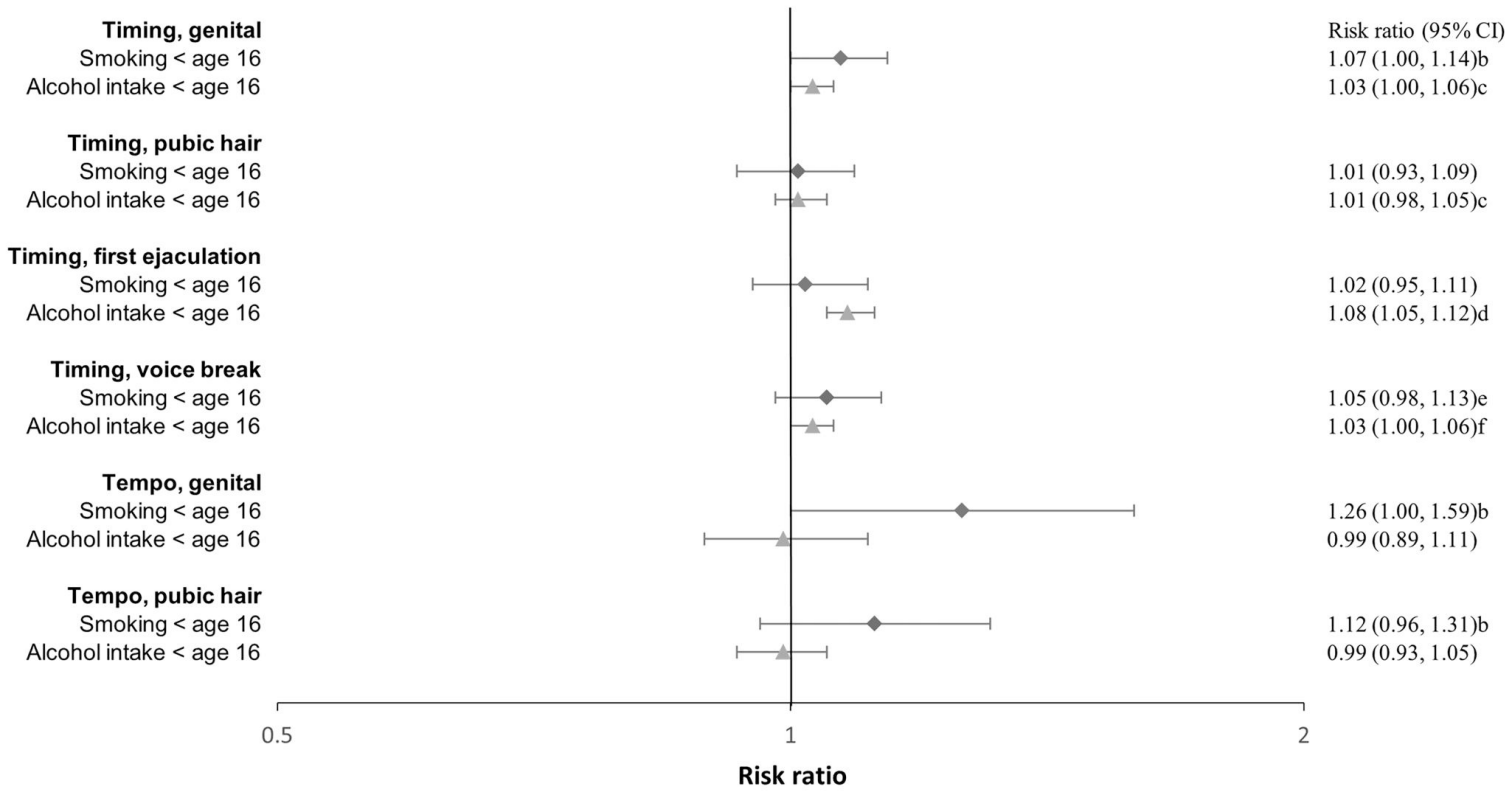
**Early debut of substance use in boys**. Age at smoking a whole cigarette or having an alcohol intake exceeding one unit across pubertal developmental groups. Adjusted^a^ risk ratios with 95% confidence interval of debut < age 16 compared to debut ≥ age 16. The estimates represent the risk ratios per year earlier pubertal development. Estimates with 95% confidence intervals are provided in the right column. ^a^Adjusted for maternal alcohol intake in pregnancy, maternal smoking in pregnancy, parental socioeconomic status in pregnancy, parental cohabitation in childhood, childhood internalizing and externalizing symptoms and behaviours, parental smoking in childhood (yes/no), and parental alcohol intake in childhood (yes/no). ^b^Not adjusted for parental alcohol intake in childhood. ^c^Not adjusted for parental smoking in childhood. ^d^Not adjusted for internalizing symptoms, cohabitation in childhood and maternal smoking in pregnancy. ^e^Not adjusted for internalizing symptoms and cohabitation in childhood. ^f^Not adjusted for internalizing symptoms, parental alcohol intake and smoking in childhood, and cohabitation.

In both girls and boys, an earlier age at pubertal timing was associated with a higher risk of smoking at age 18 (Figs 2 and 3, Supplementary Tables S4 and S5) and ever having tried to smoke a whole cigarette (Figs 4 and 5, Supplementary Tables S6 and S7); however, the association for pubic hair was less clear. Among participants who had tried smoking a whole cigarette, 42% of girls and 41% of boys had done so before the age of 16 years. In girls, earlier age at pubertal timing was associated with higher risk of smoking the first cigarette for the first time before age 16 compared to after. In boys, this was observed for genital timing and voice break (Figs 6 and 7, Supplementary Tables S8 and S9).

A faster pubertal tempo also tended to be associated with a higher risk of smoking daily or weekly at age 18 in both girls and boys, ever having tried to smoke a whole cigarette in girls, and having had a smoking debut before age 16 years in both girls and boys. Given the wide confidence intervals, the findings for tempo may also be compatible with no association (Figs 2, 3, 4, 5, 6, and 7 and Supplementary Tables S4, S5, S6, S7, S8, and S9).

### Alcohol

In girls, earlier age at menarche was associated with a lower risk of alcohol consumption at age 18 years (Fig. 2, Supplementary Table S4). In contrast, earlier age at pubertal development in boys was associated with a higher risk of drinking alcohol at age 18 (Fig. 3, Supplementary Table S5). Among participants who had consumed more than one alcohol unit, 73% of girls and 69% of boys reported doing so before the age of 16 years. In both girls and boys, an earlier age at pubertal development was associated with consuming more than one alcohol unit for the first time before age 16, compared to after. For boys, the findings were less consistent for the timing of pubic hair (Figs 6 and 7, Supplementary Tables S8 and S9). In girls, faster pubertal tempo tended to be associated with a higher risk of weekly alcohol consumption and alcohol consumption at an age <16 years (Figs 2 and 6, Supplementary Tables S4 and S8). In boys, no overall patterns were observed for tempo (Figs 3 and 7, Supplementary Tables S5 and S9).

### Recreational drug use

In both girls and boys, earlier timing of puberty was associated with a higher risk of recreational drug use at age 18 years; however, in girls, the findings mainly related to monthly drug use (Figs 2 and 3, Supplementary Tables S4 and S5). An earlier timing of pubertal development was also associated with having ever used recreational drugs compared to not having tried (Figs 4 and 5, Supplementary Tables S6 and S7). In girls, a faster tempo of pubic hair development also tended to be associated with a higher risk of recreational drug use at age 18 years and having tried using drugs (Figs 2 and 4, Supplementary Tables S4 and S6). This was not observed for tempo in boys (Figs 3 and 5, Supplementary Tables S5 and S7).

An additional analysis including only hash or pot use also showed associations between earlier age at pubertal timing and use of hash or pot (Supplementary Tables S10 and S11).

## Discussion

We found that earlier age at pubertal development was associated with higher risk of smoking cigarettes and using recreational drugs and, for boys, also with alcohol consumption at age 18 years. Furthermore, participants who experienced earlier development were more likely to have tried smoking or using recreational drugs, and have a debut of smoking or drinking alcohol before the age of 16 years. The findings for pubertal tempo were less consistent; however, a faster tempo tended to be associated with a higher risk of smoking at age 18 years for both sexes and, for girls, also the risk of drinking alcohol and using drugs.

### Strengths

In this large cohort, the detailed information on current pubertal development, collected longitudinally throughout puberty, allowed for an extensive assessment of each individual’s pubertal development, capturing not only the timing but also the tempo of Tanner stage progression, with a limited risk of recall bias. Having data on pubertal development in both girls and boys was a major strength. Registration of cigarette smoking, alcohol consumption, and drug use at the age of 18 years (after puberty) and restriction to participants who had not started smoking or drinking alcohol at the age of 10 years for girls and 11 years for boys minimized the risk of reverse causation. Socioeconomic status, maternal smoking, and impaired childhood psychological well-being are all factors that have been associated with the timing of puberty and further associated with substance use (Parent *et al.*, 2003; Deardorff *et al.*, 2014; Marshall, 2014; Wellman *et al.*, 2016; Brix *et al.*, 2019b). We were able to account for these factors in our analyses, thereby limiting the risk of confounding, which is another important strength of this study. Due to the risk of over-adjustment, analyses of the different outcomes were not mutually adjusted.

### Limitations

To be included in the final study population, participants had to have a parent who responded to the DNBC 7-year questionnaire and, themselves, have been invited and responded to the questionnaires in the Puberty Cohort, and finally have responded to DNBC-18. This selection process constitutes a risk of selection bias if smoking/alcohol/drug habits at 18 years of age are associated with participation in each of the follow-up waves. Attempting to account for this, we applied selection weights. We conducted several analyses, which introduced a risk of chance findings due to multiple testing. Information on pubertal development was not obtained by clinical examinations but was self-reported by the children, contributing to an impressive participation rate in the Puberty Cohort of 70%. A validation study confirmed that the self-reported information in the Puberty Cohort was reliable and could be used for etiological studies (Ernst *et al.*, 2018). For some milestones, an exact age was not provided and was imputed under the assumption that the missing data were missing at random. Including other pubertal milestones and a broad set of covariates in the imputation model helped make this assumption more plausible. Information about cigarette smoking, alcohol consumption, and drug use was also self-reported as part of a comprehensive questionnaire that included questions on various topics such as lifestyle and health. A potential underreporting of the outcomes is possible due to social stigmatization; however, the proportion of smoking participants was in line with the proportion of young smokers observed in another Danish study with information on smoking habits among 15- to 29-year-olds in 2020 (Bast *et al.*, 2022). Similar to the child’s smoking and alcohol consumption habits, the parent’s habits may be prone to under-reporting, leading to a risk of residual confounding.

Over the past few years, several new nicotine and tobacco products have been introduced. In the DNBC-18 questionnaire, participants were only asked to report cigarette and e-cigarette use, and due to a limited number of e-cigarette users, we were not able to investigate this use. The Danish study on smoking habits reported that cigarettes were typically the first product used, supporting the relevance of investigating cigarette use in this young cohort (Bast *et al.*, 2022).

### Interpretations

In this study, information on several physical signs of puberty was investigated, allowing for investigation of milestone-specific effects within the same cohort. While most pubertal milestones are driven by activation of the hypothalamic–pituitary–gonadal (HPG) axis, the development of acne, pubic hair, and axillary hair is primarily influenced by adrenarche, where increased levels of androgens are secreted by the adrenal gland (Wood *et al.*, 2019). For most of the outcomes investigated, the results pointed in the same direction across pubertal milestones. Some findings, however, were less pronounced for pubic hair development, raising the possibility that adrenarche may play a different role in the risk of substance use. Previous studies on substance use have mainly focused on milestones related to HPG-axis activation; however, including markers of adrenarche in future studies may provide additional insights, given its proposed influence on brain development and associations between earlier adrenarche and mental health problems (Byrne *et al.*, 2017). In contrast to studies using physical measures of puberty, some studies show that self-perceived earlier pubertal development relative to peers is also associated with substance use. In such cases, pubertal development is viewed in a social context, emphasizing the impact of being off-time compared to peers (Bratberg *et al.*, 2007; Kong *et al.*, 2013). We investigated substance use at age 18 and found consistent associations, although some studies suggest an even stronger association with substance use when assessed at earlier time points (Kaltiala-Heino *et al.*, 2011; Cance *et al.*, 2013). In contrast, a US study investigated the long-term consequences of early puberty on substance use and found associations with cannabis use also in adulthood (Goering *et al.*, 2024).

The association between early pubertal development and substance use is suggested to be caused by socializing with older peers and thus being introduced to cigarettes, alcohol, or recreational drugs at an earlier age. Moreover, entering a vulnerable phase in life before being cognitively developed to resist risk-taking behaviours might increase the risk (Lee *et al.*, 2014). Another theory, referred to as the *off-time theory*, suggests that being at a different maturity level compared to peers induces emotional stress that increases vulnerability to external exposures (Graber, 2013). In our study, the observed higher risk of smoking, drinking alcohol (boys), and using drugs at the age of 18 years among those who experienced an earlier pubertal development could potentially be explained by an extended period at risk, given that puberty is a vulnerable period. A study on pubertal boys scanned by functional magnetic resonance imaging found that brain responses to risky decisions vary by pubertal stage, suggesting reduced negative feedback and a higher risk of engaging in risk-taking behaviours in puberty (Goddings *et al.*, 2023). A cross-sectional study conducted in the USA and Australia found that having a more advanced stage of pubertal development was associated with a higher risk of substance use, also after controlling for age (Patton *et al.*, 2004). We investigated the risk of substance use among young men and women, among whom the majority had completed puberty and were at the same developmental level. This indicates that not only the pubertal stage but also the timing is important for the risk of starting and continuing to smoke, drink alcohol, and use recreational drugs. To further address the risk that participants may have quit their use before our follow-up at age 18, we investigated ‘ever’ substance use, and the analyses supported that early developers were more willing to try cigarettes and recreational drugs. Among smokers and alcohol consumers at age 18, we found that participants with an earlier pubertal development had a higher risk of initiating use before age 16. These findings are concerning, as research shows that the negative health effects of smoking worsen with prolonged duration (Le Foll *et al.*, 2022), and an early smoking debut is associated with an increased risk of nicotine dependence and a lower success of smoking cessation (Khuder *et al.*, 1999; Mittal *et al.*, 2024). Additionally, starting to drink alcohol at an early age may increase the risk of later alcohol use disorders (Blomeyer *et al.*, 2013).

A US study from 1994 investigated the tempo of puberty in relation to cigarette smoking and alcohol consumption among girls. A faster tempo was associated with having a younger age at moderate alcohol consumption, whereas no association was reported with cigarette smoking (Wilson *et al.*, 1994). In contrast, the results from our study indicated an association between pubertal tempo and both cigarette smoking and alcohol consumption in girls. A correlation between earlier timing and faster tempo of puberty has been described (Lunddorf *et al.*, 2024), and our findings of an increased risk with a faster tempo could, to some degree, be explained by an earlier age, and vice versa. Due to sparse data, we were unable to investigate interactions between timing and tempo in this study.

In conclusion, we found that girls and boys with earlier pubertal timing, and possibly also those with faster tempo, were more likely to report substance use during adolescence, including early initiation of smoking and alcohol use. Further research may help clarify the underlying pathways and mechanisms and explore whether interventions targeting individuals with early pubertal timing can reduce adolescent substance use and delay its onset.

## Supplementary Material

hoaf072_Supplementary_Data

## Data Availability

The data that support the findings of this study are available from the Danish National Birth Cohort. Restrictions apply to the availability of these data, which were used under license for this study. Data are available after permission from the Danish National Birth Cohort. The procedure for applying for data access is available on the website: https://www.dnbc.dk/access-to-dnbc-data.
