# Supplementary material for: Timing and tempo of pubertal development and substance use in adolescence: a cohort study in the Danish National Birth Cohort
Source: Hum Reprod Open. 2025 Nov 18;2025(4):hoaf072. doi: 10.1093/hropen/hoaf072 (PMC12976676; doi:10.1093/hropen/hoaf072)
Supplement: hoaf072_Supplementary_Data [file hoaf072_Supplementary_Data.zip › Supplementary-file-S1-post adjudication clean.docx]

**Supplementary File S1**: Selection weights.

To be included in the final study population, participants had to have participated in the 7-year questionnaire, the Puberty Cohort, and the DNBC-18 questionnaire. Therefore, we calculated three selection weights that were multiplied and further multiplied with the sampling weights to obtain a single weight for each participant. The selection weights were calculated as 1 divided by the probability of participating, and the probability was calculated using a multivariable logistic regression model including variables assumed to affect the willingness to participate. The first weight was calculated among the 22,439 who were invited to the Puberty Cohort as their probability of participating in the 7-year questionnaire. Among those who participated in the 7-year questionnaire, we calculated the second weight as their probability of participating in the Puberty Cohort (for analyses on Tanner stages we calculated their probability of participating with at least two questionnaires). Among those who also participated in the Puberty Cohort, we calculated the third weight as their probability of participating in the 18-year follow-up.
