# Supplementary material for: Timing and tempo of pubertal development and substance use in adolescence: a cohort study in the Danish National Birth Cohort
Source: Hum Reprod Open. 2025 Nov 18;2025(4):hoaf072. doi: 10.1093/hropen/hoaf072 (PMC12976676; doi:10.1093/hropen/hoaf072)
Supplement: hoaf072_Supplementary_Data [file hoaf072_Supplementary_Data.zip › Supplementary-tables-post_adjudication_clean_EO.docx]

**Supplementary Table S1:** Pseudo range^a^, mean and SD of the different categories for pubertal development among 4,935 girls and 3,128 boys eligible for analyses on Tanner stages, and 5,554 girls eligible for analyses on menarche and 3,625 boys eligible for analyses on first ejaculation and voice break.

|  | Timing (age in years) | | | Tempo (Tanner stages per year) | | |
| --- | --- | --- | --- | --- | --- | --- |
|  | Earlier (15%) | Average (70%) | Later (15%) | Faster (15%) | Average (70%) | Slower (15%) |
| **Girls** |  |  |  |  |  |  |
| **Breast** |  |  |  |  |  |  |
| Mean (SD) | 10.6 (0.5) | 12.3 (0.7) | 14.2 (0.6) | 1.1 (0.1) | 0.7 (0.2) | 0.3 (0.1) |
| Pseudo range | 9.2 – 11.1 | 11.1 – 13.5 | 13.5 – 17.0 | 1.0 – 1.6 | 0.4 – 1.0 | 0.0 – 0.4 |
| **Pubic hair** |  |  |  |  |  |  |
| Mean (SD) | 11.4 (0.4) | 12.9 (0.6) | 14.7 (0.6) | 1.6 (0.1 | 1.0 (0.2) | 0.5 (0.2) |
| Pseudo range | 9.9 – 11.9 | 11.9 – 14.0 | 14.0 – 17.5 | 1.4 – 2.1 | 0.7 – 1.4 | -0.7 – 0.7 |
| **Menarche** |  |  |  |  |  |  |
| Mean (SD) | 11.3 (0.4) | 12.9 (0.6) | 14.8 (0.6) | - | - | - |
| Pseudo range | 8.2 – 11.8 | 11.8 – 14.2 | 14.2 – 18.6 | - | - | - |
| **Boys** |  |  |  |  |  |  |
| **Genitals** |  |  |  |  |  |  |
| Mean (SD) | 11.4 (0.5) | 13.0 (0.6) | 14.9 (0.6) | 1.4 (0.1) | 0.9 (0.2) | 0.4 (0.2) |
| Pseudo range | 9.7 – 11.9 | 11.9 – 14.2 | 14.2 – 17.3 | 1.2 – 1.9 | 0.5 – 1.2 | -0.7 – 0.5 |
| **Pubic hair** |  |  |  |  |  |  |
| Mean (SD) | 11.7 (0.4) | 13.0 (0.5) | 14.7 (0.5) | 2.1 (0.2) | 1.3 (0.3) | 0.6 (0.3) |
| Pseudo range | 10.2 – 12.1 | 12.1 – 14.1 | 14.1 – 16.8 | 1.9 – 2.8 | 0.8 – 1.9 | -0.9 – 0.8 |
| **First ejaculation** |  |  |  |  |  |  |
| Mean (SD) | 11.2 (0.7) | 13.2 (0.7) | 15.4 (0.8) | - | - | - |
| Pseudo range | 5.4 – 12.0 | 12.0 – 14.5 | 14.5 – 19.7 | - | - | - |
| **Voice break** |  |  |  |  |  |  |
| Mean (SD) | 10.9 (0.6) | 13.0 (0.7) | 15.1 (0.7) | - | - | - |
| Pseudo range | 7.3 – 11.6 | 11.6 – 14.3 | 14.3 – 19.5 | - | - | - |

^a^The pseudo range is the mean of five observations closest to the actual range. It is provided to avoid showing individual-level observations and to meet the General Data Protection Regulation (GDPR, Regulation (EU), 2016/679 of May 25, 2018).

**Supplementary Table S2**: Covariates included in the imputation model used to obtain a specific age at menarche, first ejaculation, and voice break.

| Pregnancy | Parental socioeconomic status, parental cohabitation, maternal smoking, maternal alcohol use, maternal BMI, maternal worries, any maternal mental disorder, and self-reported maternal age at menarche |
| --- | --- |
| Age 7 | The child's externalizing and internalizing symptoms, childhood BMI, parental cohabitation, maternal smoking, paternal smoking, maternal alcohol intake, paternal alcohol intake, and maternal psychiatric disorders since birth |
| Puberty | In the model for girls: timing and tempo of pubic hair and breast development, and age at menarche  In the model for boys: timing and tempo of pubic hair and genital development, and age at first ejaculation and voice break |
| Age 18 | The participants' current smoking, alcohol intake, and drug use |
| Other covariates from registers | Parity and maternal age |

**Supplementary Table S3:** Different adjustment strategies for the association between genital timing and the risk of ever having tried to smoke a cigarette. Risk ratios (RR) with 95% confidence interval.

|  | Crude | Model 1: RR (95% CI)^a^ | Model 2: RR (95% CI)^b^ | Model 3: RR (95% CI)^c^ |
| --- | --- | --- | --- | --- |
| **Genital timing** |  |  |  |  |
| Earlier | 1.11 | 1.06 (0.93, 1.22) | 1.07 (0.93, 1.23) | 1.06 (0.92, 1.22) |
| Average |  |  |  |  |
| Later | 0.75 | 0.77 (0.63, 0.92) | 0.77 (0.64, 0.93) | 0.77 (0.64, 0.92) |
| Continous model | 1.10 | 1.08 (1.03, 1.13) | 1.08 (1.04, 1.13) | 1.08 (1.03, 1.13) |

^a^Adjusted for maternal alcohol intake in pregnancy, maternal smoking in pregnancy, parental socioeconomic status in pregnancy, parental cohabitation in childhood, childhood internalizing and externalizing symptoms and behaviours, paternal and maternal smoking in childhood, paternal and maternal alcohol intake in childhood.

^b^Not adjusted for parental smoking and alcohol intake in childhood.

^c^Not adjusted for internalizing symptoms and cohabitation.

**Supplementary Table S4**: Girls' substance use at age 18 - categorical model. Relative risk ratios for smoking cigarettes, drinking alcohol, or using drugs at age 18 compared to not smoking, not drinking alcohol (≤1 time/month), or not using recreational drugs, across pubertal developmental groups.

|  | Cigarettes, weekly or daily | | Cigarettes, monthly | | Alcohol, weekly | | Alcohol, 2-4x/month | | Drugs, monthly | | Drugs, <1x/month | |
| --- | --- | --- | --- | --- | --- | --- | --- | --- | --- | --- | --- | --- |
|  | Crude | Adjusted (95% CI)^a^ | Crude | Adjusted (95% CI)^a^ | Crude | Adjusted (95% CI)^a^ | Crude | Adjusted (95% CI)^a^ | Crude | Adjusted (95% CI)^a^ | Crude | Adjusted (95% CI)^a^ |
| **Timing, breast** |  |  |  |  |  |  |  |  |  |  |  |  |
| Earlier | 1.16 | 1.06 (0.72, 1.55) | 1.24 | 1.18 (0.78, 1.78) | 0.87 | 0.88 (0.54, 1.43) | 0.83 | 0.88 (0.67, 1.14) | 1.66 | 1.46 (0.73, 2.94) | 1.17 | 1.12 (0.80, 1.56) |
| Average (ref) |  |  |  |  |  |  |  |  |  |  |  |  |
| Later | 0.57 | 0.63 (0.39, 1.02) | 0.73 | 0.74 (0.47, 1.16) | 1.29 | 1.26 (0.83, 1.91) | 0.86 | 0.82 (0.63, 1.08) | 0.27 | 0.31 (0.09, 1.01) | 1.04 | 1.07 (0.75, 1.52) |
| **Timing, pubic hair** |  |  |  |  |  |  |  |  |  |  |  |  |
| Earlier | 0.70 | 0.67 (0.44, 1.02) | 1.12 | 1.08 (0.70, 1.65) | 0.73 | 0.77 (0.47, 1.25) | 0.83 | 0.89 (0.68, 1.15) | 0.98 | 0.88 (0.38, 2.00) | 0.91 | 0.93 (0.66, 1.30) |
| Average (ref) |  |  |  |  |  |  |  |  |  |  |  |  |
| Later | 0.59 | 0.63 (0.40, 0.98) | 0.76 | 0.79 (0.52, 1.20) | 1.06 | 1.10 (0.71, 1.72) | 0.88 | 0.90 (0.69, 1.19) | 0.30 | 0.31 (0.11, 0.88) | 0.81 | 0.82 (0.57, 1.20) |
| **Timing, menarche** |  |  |  |  |  |  |  |  |  |  |  |  |
| Earlier | 1.24 | 1.08 (0.75, 1.56) | 1.32 | 1.29 (0.84, 1.98) | 0.83 | 0.88 (0.55, 1.41) | 0.76 | 0.83 (0.64, 1.08) | 1.06 | 0.99 (0.48, 2.04) | 1.13 | 1.10 (0.78, 1.55) |
| Average (ref) |  |  |  |  |  |  |  |  |  |  |  |  |
| Later | 0.50 | 0.55 (0.33, 0.91) | 0.78 | 0.81 (0.52, 1.26) | 1.46 | 1.39 (0.90, 2.15) | 1.16 | 1.08 (0.82, 1.44) | 0.20 | 0.21 (0.06, 0.71) | 1.11 | 1.13 (0.80, 1.59) |
| **Tempo, breast** |  |  |  |  |  |  |  |  |  |  |  |  |
| Faster | 1.04 | 1.05 (0.70, 1.56) | 1.11 | 1.06 (0.69, 1.61) | 1.09 | 1.12 (0.71, 1.76) | 0.96 | 1.00 (0.77, 1.30) | 1.37 | 1.33 (0.62, 2.89) | 1.03 | 0.99 (0.71, 1.39) |
| Average (ref) |  |  |  |  |  |  |  |  |  |  |  |  |
| Slower | 0.88 | 0.85 (0.55, 1.32) | 0.86 | 0.83 (0.53, 1.30) | 0.91 | 0.86 (0.55, 1.35) | 1.19 | 1.16 (0.89, 1.53) | 0.79 | 0.77 (0.32, 1.83) | 0.92 | 0.90 (0.64, 1.26) |
| **Tempo, pubic hair** |  |  |  |  |  |  |  |  |  |  |  |  |
| Faster | 0.93 | 0.89 (0.60, 1.33) | 1.25 | 1.25 (0.82, 1.92) | 0.97 | 0.99 (0.64, 1.53) | 0.84 | 0.89 (0.68, 1.17) | 0.66 | 0.58 (0.27, 1.25) | 1.16 | 1.14 (0.82, 1.57) |
| Average (ref) |  |  |  |  |  |  |  |  |  |  |  |  |
| Slower | 0.77 | 0.80 (0.51, 1.26) | 1.14 | 1.12 (0.74, 1.70) | 0.62 | 0.59 (0.37, 0.97) | 0.85 | 0.83 (0.64, 1.08) | 0.30 | 0.31 (0.12, 0.78) | 0.85 | 0.86 (0.59, 1.24) |

^a^Adjusted for maternal alcohol intake in pregnancy, maternal smoking in pregnancy, parental socioeconomic status in pregnancy, parental cohabitation in childhood, childhood internalizing and externalizing symptoms and behaviours, paternal and maternal smoking in childhood, paternal and maternal alcohol intake in childhood.

**Supplementary Table S5**: Boys' substance use at age 18 - categorical model. Relative risk ratios for smoking cigarettes, drinking alcohol, or using drugs at age 18 compared to not smoking, not drinking alcohol (≤1 time/month), or not using recreational drugs, across pubertal developmental groups.

|  | Cigarettes, weekly or daily | | Cigarettes, monthly | | Alcohol, weekly | | Alcohol, 2-4x/month | | Drugs, monthly | | Drugs, <1x/month | |
| --- | --- | --- | --- | --- | --- | --- | --- | --- | --- | --- | --- | --- |
|  | Crude | Adjusted (95% CI)^a^ | Crude | Adjusted (95% CI)^a^ | Crude | Adjusted (95% CI)^a^ | Crude | Adjusted (95% CI)^a^ | Crude | Adjusted (95% CI)^a^ | Crude | Adjusted (95% CI)^a^ |
| **Timing, genital** |  |  |  |  |  |  |  |  |  |  |  |  |
| Earlier | 1.06 | 0.86 (0.54, 1.38) | 0.94 | 0.93 (0.52, 1.65) | 1.20 | 1.20 (0.77, 1.88) | 1.26 | 1.28 (0.89, 1.84) | 1.46 | 1.33 (0.78, 2.30) | 1.17 | 1.17 (0.80, 1.70) |
| Average (ref) |  |  |  |  |  |  |  |  |  |  |  |  |
| Later | 0.56 | 0.64 (0.35, 1.17) | 0.35 | 0.35 (0.19, 0.67) | 0.74 | 0.73 (0.46, 1.17) | 1.03 | 1.01 (0.72, 1.43) | 0.98 | 1.05 (0.56, 1.96) | 0.68 | 0.66 (0.43, 1.01) |
| **Timing, pubic hair** |  |  |  |  |  |  |  |  |  |  |  |  |
| Earlier | 0.85 | 0.79 (0.49, 1.30) | 0.66 | 0.65 (0.39, 1.09) | 1.21 | 1.27 (0.83, 1.95) | 0.92 | 0.92 (0.65, 1.29) | 0.79 | 0.71 (0.42, 1.21) | 0.83 | 0.81 (0.56, 1.17) |
| Average (ref) |  |  |  |  |  |  |  |  |  |  |  |  |
| Later | 0.89 | 1.01 (0.62, 1.67) | 0.49 | 0.48 (0.26, 0.88) | 1.09 | 1.04 (0.66, 1.63) | 1.13 | 1.10 (0.78, 1.56) | 0.66 | 0.66 (0.35, 1.24) | 0.61 | 0.57 (0.38, 0.87) |
| **Timing, first ejaculation** |  |  |  |  |  |  |  |  |  |  |  |  |
| Earlier | 1.43 | 1.34 (0.84, 2.12) | 1.05 | 1.04 (0.62, 1.73) | 1.51 | 1.52 (0.97, 2.36) | 1.08 | 1.07 (0.74, 1.57) | 1.69 | 1.65 (0.95, 2.88) | 1.30 | 1.28 (0.89, 1.84) |
| Average (ref) |  |  |  |  |  |  |  |  |  |  |  |  |
| Later | 0.77 | 0.79 (0.45, 1.38) | 0.78 | 0.78 (0.41, 1.48) | 0.64 | 0.65 (0.39, 1.07) | 0.74 | 0.76 (0.53, 1.08) | 0.60 | 0.60 (0.29, 1.23) | 0.63 | 0.63 (0.39, 1.02) |
| **Timing, voice break** |  |  |  |  |  |  |  |  |  |  |  |  |
| Earlier | 0.99 | 0.91 (0.53, 1.56) | 1.58 | 1.59 (0.94, 2.69) | 1.57 | 1.59 (0.99, 2.55) | 1.47 | 1.49 (1.00, 2.22) | 1.67 | 1.57 (0.89, 2.79) | 1.54 | 1.53 (1.03, 2.29) |
| Average (ref) |  |  |  |  |  |  |  |  |  |  |  |  |
| Later | 0.66 | 0.72 (0.39, 1.34) | 0.67 | 0.69 (0.34, 1.40) | 0.78 | 0.80 (0.47, 1.36) | 1.08 | 1.07 (0.73, 1.58) | 1.00 | 1.06 (0.51, 2.21) | 0.72 | 0.71 (0.45, 1.13) |
| **Tempo, genital** |  |  |  |  |  |  |  |  |  |  |  |  |
| Faster | 0.76 | 0.72 (0.44, 1.19) | 0.91 | 0.87 (0.51, 1.49) | 1.06 | 1.08 (0.69, 1.69) | 0.93 | 0.98 (0.68, 1.40) | 0.69 | 0.65 (0.34, 1.25) | 0.75 | 0.75 (0.51, 1.11) |
| Average (ref) |  |  |  |  |  |  |  |  |  |  |  |  |
| Slower | 0.38 | 0.36 (0.19, 0.68) | 0.73 | 0.76 (0.44, 1.31) | 1.02 | 1.06 (0.68, 1.67) | 0.97 | 0.97 (0.69, 1.38) | 0.47 | 0.50 (0.25, 1.00) | 0.86 | 0.85 (0.58, 1.25) |
| **Tempo, pubic hair** |  |  |  |  |  |  |  |  |  |  |  |  |
| Faster | 1.14 | 1.14 (0.69, 1.88) | 0.75 | 0.74 (0.39, 1.41) | 0.72 | 0.77 (0.49, 1.21) | 0.81 | 0.86 (0.61, 1.20) | 1.17 | 1.25 (0.68, 2.30) | 0.69 | 0.73 (0.50, 1.07) |
| Average (ref) |  |  |  |  |  |  |  |  |  |  |  |  |
| Slower | 0.85 | 0.92 (0.55, 1.54) | 0.98 | 0.99 (0.60, 1.63) | 0.88 | 0.86 (0.54, 1.38) | 1.15 | 1.11 (0.78, 1.58) | 0.71 | 0.70 (0.39, 1.27) | 0.91 | 0.86 (0.59, 1.25) |

^a^Adjusted for maternal alcohol intake in pregnancy, maternal smoking in pregnancy, parental socioeconomic status in pregnancy, parental cohabitation in childhood, childhood internalizing and externalizing symptoms and behaviours, paternal and maternal smoking in childhood, paternal and maternal alcohol intake in childhood.

**Supplementary Table S6**: Ever having used substances in girls - categorical model. Risk ratios of ever having tried smoking a whole cigarette or using drugs compared to not having tried across pubertal developmental groups.

|  | Smoking a whole  cigarette (ever) | | Recreational drug use (ever) | |
| --- | --- | --- | --- | --- |
|  | Crude | Adjusted (95% CI)^a^ | Crude | Adjusted (95% CI)^a^ |
| **Timing, breast** |  |  |  |  |
| Earlier | 1.11 | 1.08 (0.97, 1.20) | 1.22 | 1.16 (0.96, 1.40) |
| Average (ref) |  |  |  |  |
| Later | 0.83 | 0.87 (0.76, 0.99) | 0.86 | 0.89 (0.71, 1.12) |
| **Timing, pubic hair** |  |  |  |  |
| Earlier | 0.95 | 0.95 (0.85, 1.07) | 0.93 | 0.92 (0.75, 1.13) |
| Average (ref) |  |  |  |  |
| Later | 0.88 | 0.91 (0.80, 1.03) | 0.68 | 0.70 (0.54, 0.89) |
| **Timing, menarche** |  |  |  |  |
| Earlier | 1.04 | 1.01 (090, 1.13) | 1.13 | 1.09 (0.89, 1.32 |
| Average (ref) |  |  |  |  |
| Later | 0.86 | 0.89 (0.78, 1.02) | 0.94 | 0.99 (0.80, 1.22) |
| **Tempo, breast** |  |  |  |  |
| Faster | 1.01 | 1.00 (0.89, 1.12) | 1.13 | 1.08 (0.88, 1.32) |
| Average (ref) |  |  |  |  |
| Slower | 0.94 | 0.93 (0.83, 1.06) | 0.96 | 0.96 (0.78, 1.18) |
| **Tempo, pubic hair** |  |  |  |  |
| Faster | 1.04 | 1.02 (0.91, 1.15) | 1.03 | 1.02 (0.77, 1.35) |
| Average (ref) |  |  |  |  |
| Slower | 0.91 | 0.92 (0.81, 1.05) | 0.86 | 0.82 (0.61, 1.10) |

^a^Adjusted for maternal alcohol intake in pregnancy, maternal smoking in pregnancy, parental socioeconomic status in pregnancy, parental cohabitation in childhood, childhood internalizing and externalizing symptoms and behaviours, paternal and maternal smoking in childhood, paternal and maternal alcohol intake in childhood.

**Supplementary Table S7**: Ever having used substances in boys - categorical model. Risk ratios of ever having tried smoking a whole cigarette or using drugs compared to not having tried across pubertal developmental groups.

|  | Smoking a whole  cigarette (ever) | | Drug use (ever) | |
| --- | --- | --- | --- | --- |
|  | Crude | Adjusted (95% CI)^a^ | Crude | Adjusted (95% CI)^a^ |
| **Timing, genital** |  |  |  |  |
| Earlier | 1.11 | 1.06 (0.93, 1.22) | 1.17 | 1.16 (0.99, 1.35) |
| Average (ref) |  |  |  |  |
| Later | 0.75 | 0.77 (0.63, 0.92) | 0.80 | 0.80 (0.64, 1.00) |
| **Timing, pubic hair** |  |  |  |  |
| Earlier | 0.93 | 0.91 (0.78, 1.06) | 0.96 | 0.93 (0.77, 1.11) |
| Average (ref) |  |  |  |  |
| Later | 0.81 | 0.83 (0.70, 0.98) | 0.72 | 0.72 (0.58, 0.90) |
| **Timing, first ejaculation** |  |  |  |  |
| Earlier | 1.10 | 1.07 (0.95, 1.25) | 1.16 | 1.19 (1.03, 1.39)^b^ |
| Average (ref) |  |  |  |  |
| Later | 0.75 | 0.76 (0.62, 0.92) | 0.72 | 0.74 (0.58, 0.94)^b^ |
| **Timing, voice break** |  |  |  |  |
| Earlier | 1.21 | 1.17 (1.02, 1.34) | 1.25 | 1.23 (1.04, 1.45) |
| Average (ref) |  |  |  |  |
| Later | 0.77 | 0.78 (0.63, 0.96) | 0.82 | 0.83 (0.65, 1.06) |
| **Tempo, genital** |  |  |  |  |
| Faster | 0.91 | 0.90 (0.77, 1.06) | 0.86 | 0.87 (0.71, 1.05) |
| Average (ref) |  |  |  |  |
| Slower | 0.93 | 0.93 (0.79, 1.08) | 0.82 | 0.86 (0.70, 1.05) |
| **Tempo, pubic hair** |  |  |  |  |
| Faster | 0.90 | 0.88 (0.75, 1.03) | 0.84 | 0.88 (0.73, 1.08) |
| Average (ref) |  |  |  |  |
| Slower | 0.99 | 1.02 (0.89, 1.18) | 0.93 | 0.94 (0.79, 1.13) |

^a^Adjusted for maternal alcohol intake in pregnancy, maternal smoking in pregnancy, parental socioeconomic status in pregnancy, parental cohabitation in childhood, childhood internalizing and externalizing symptoms and behaviours, paternal and maternal smoking in childhood, paternal and maternal alcohol intake in childhood.

^b^Not adjusted for parental alcohol intake in childhood.

|  |  | Smoking a whole cigarette < age 16 |  | Alcohol intake exceeding one unit < age 16 |
| --- | --- | --- | --- | --- |
|  | Crude | Adjusted (95% CI)^a^ | Crude | Adjusted (95% CI)^a^ |
| **Timing, breast** |  |  |  |  |
| Earlier | 1.10 | 1.05 (0.89, 1.25) | 1.02 | 1.01 (0.95, 1.08) |
| Average (ref) |  |  |  |  |
| Later | 0.82 | 0.86 (0.68, 1.08) | 0.91 | 0.91 (0.84, 0.99) |
| **Timing, pubic hair** |  |  |  |  |
| Earlier | 1.06 | 1.05 (0.88, 1.25) | 1.00 | 1.01 (0.95, 1.08) |
| Average (ref) |  |  |  |  |
| Later | 0.87 | 0.88 (0.70, 1.10) | 0.93 | 0.93 (0.86, 1.00) |
| **Timing, menarche** |  |  |  |  |
| Earlier | 1.18 | 1.14 (0.97, 1.35) | 1.07 | 1.05 (0.99, 1.12)^b^ |
| Average (ref) |  |  |  |  |
| Later | 0.82 | 0.85 (0.67, 1.08) | 0.93 | 0.93 (0.86, 1.00)^b^ |
| **Tempo, breast** |  |  |  |  |
| Faster | 1.14 | 1.11 (0.93, 1.32) | 1.07 | 1.06 (1.00, 1.12) |
| Average (ref) |  |  |  |  |
| Slower | 0.90 | 0.89 (0.72, 1.10) | 1.00 | 1.02 (0.95, 1.08) |
| **Tempo, pubic hair** |  |  |  |  |
| Faster | 1.04 | 1.01 (0.83, 1.21) | 1.04 | 1.04 (0.98, 1.10) |
| Average (ref) |  |  |  |  |
| Slower | 0.81 | 0.82 (0.65, 1.03) | 1.01 | 1.01 (0.95, 1.08) |

**Supplementary Table S8**: Early debut of substance use in girls - categorical analysis. Age at smoking a whole cigarette or having an alcohol intake exceeding one unit across pubertal developmental groups. Risk ratios of debut < age 16 compared to debut ≥ age 16.

^a^Adjusted for maternal alcohol intake in pregnancy, maternal smoking in pregnancy, parental socioeconomic status in pregnancy, parental cohabitation in childhood, childhood internalizing and externalizing symptoms and behaviours, parental smoking in childhood (yes/no), parental alcohol intake in childhood (yes/no).

^b^Not adjusted for parental smoking in childhood.

**Supplementary Table S9**: Early debut of substance use in boys categorical model. Age at smoking a whole cigarette or having an alcohol intake exceeding one unit across pubertal developmental groups. Risk ratios of debut < age 16 compared to debut ≥ age 16.

|  |  | Smoking a whole cigarette < age 16 |  | Alcohol intake exceeding one unit < age 16 |
| --- | --- | --- | --- | --- |
|  | Crude | Adjusted (95% CI)^a^ | Crude | Adjusted (95% CI)^a^ |
| **Timing, genital** |  |  |  |  |
| Earlier | 1.26 | 1.14 (0.96, 1.36)^b^ | 1.04 | 1.03 (0.95, 1.12) |
| Average (ref) |  |  |  |  |
| Later | 0.75 | 0.76 (0.57, 1.03)^b^ | 0.89 | 0.90 (0.80, 1.00) |
| **Timing, pubic hair** |  |  |  |  |
| Earlier | 1.06 | 1.05 (0.85, 1.29) | 0.94 | 0.92 (0.82, 1.03) |
| Average (ref) |  |  |  |  |
| Later | 0.88 | 0.89 (0.70, 1.14) | 0.94 | 0.96 (0.87, 1.05) |
| **Timing, first ejaculation** |  |  |  |  |
| Earlier | 1.17 | 1.07 (0.83, 1.36)^b^ | 1.06 | 1.06 (0.97, 1.16)^c^ |
| Average (ref) |  |  |  |  |
| Later | 0.88 | 0.89 (0.67, 1.17)^b^ | 0.82 | 0.82 (0.72, 0.93)^c^ |
| **Timing, voice break** |  |  |  |  |
| Earlier | 1.07 | 1.13 (0.89, 1.43)^c^ | 1.05 | 1.04 (0.95, 1.15)^c^ |
| Average (ref) |  |  |  |  |
| Later | 0.93 | 0.98 (0.73, 1.31)^c^ | 0.88 | 0.89 (0.77, 1.02)^c^ |
| **Tempo, genital** |  |  |  |  |
| Faster | 1.16 | 1.24 (0.81, 1.90)^d^ | 0.99 | 1.00 (0.93, 1.08) |
| Average (ref) |  |  |  |  |
| Slower | 0.68 | 0.60 (0.38, 0.95)^d^ | 0.96 | 0.93 (0.82, 1.06) |
| **Tempo, pubic hair** |  |  |  |  |
| Faster | 1.11 | 1.09 (0.90, 1.33) | 1.01 | 0.99 (0.89, 1.09) |
| Average (ref) |  |  |  |  |
| Slower | 0.88 | 0.89 (0.70, 1.15) | 1.00 | 0.98 (0.89, 1.09) |

^a^Adjusted for maternal alcohol intake in pregnancy, maternal smoking in pregnancy, parental socioeconomic status in pregnancy, parental cohabitation in childhood, childhood internalizing and externalizing symptoms and behaviours, parental smoking in childhood (yes/no), parental alcohol intake in childhood (yes/no).

^b^Not adjusted for parental smoking and alcohol intake in childhood.

^c^Not adjusted for internalizing symptoms and cohabitation in childhood.

^d^Not adjusted for parental alcohol intake in childhood.

**Supplementary Table S10:** Girls' hash or pot use at age 18. Relative risk ratios for using hash or pot at age 18 compared to no use across pubertal developmental groups.

|  | Hash or pot, monthly | | Hash or pot, <1x/month | |
| --- | --- | --- | --- | --- |
|  | Crude | Adjusted (95% CI)^a^ | Crude | Adjusted (95% CI)^a^ |
| **Timing, breast** |  |  |  |  |
| Earlier | 1.49 | 1.31 (0.60, 2.85) | 1.12 | 1.09 (0.77, 1.54) |
| Average (ref) |  |  |  |  |
| Later | 0.24 | 0.27 (0.07, 1.05) | 0.95 | 0.98 (0.68, 1.40) |
| Continuous model (per year earlier) | 1.36 | 1.27 (1.03, 1.56) | 1.07 | 1.07 (0.96, 1.18) |
| **Timing, pubic hair** |  |  |  |  |
| Earlier | 1.01 | 0.93 (0.68, 1.34) | 0.89 | 0.91 (0.64, 1.28) |
| Average (ref) |  |  |  |  |
| Later | 0.29 | 0.30 (0.09, 0.96) | 0.71 | 0.71 (0.48, 1.05) |
| Continuous model (per year earlier) | 1.20 | 1.15 (0.91, 1.45) | 1.13 | 1.13 (1.01, 1.27) |
| **Timing, menarche** |  |  |  |  |
| Earlier | 0.99 | 0.93 (0.42, 2.07) | 1.14 | 1.13 (0.80, 1.60) |
| Average (ref) |  |  |  |  |
| Later | 0.21 | 0.22 (0.06, 0.75) | 1.04 | 1.06 (0.74, 1.51) |
| Continuous model (per year earlier) | 1.35 | 1.29 (1.02, 1.63) | 1.05 | 1.04 (0.94, 1.17) |
| **Tempo, breast** |  |  |  |  |
| Faster | 1.47 | 1.41 (0.63, 3.18) | 1.12 | 1.07 (0.76, 1.50) |
| Average (ref) |  |  |  |  |
| Slower | 0.92 | 0.89 (0.37, 2.17) | 0.95 | 0.94 (0.67, 1.33) |
| Continuous model (Tanner stages/year) | 0.99 | 0.78 (0.20, 3.05) | 1.41 | 1.34 (0.85, 2.13) |
| **Tempo, pubic hair** |  |  |  |  |
| Faster | 0.66 | 0.58 (0.26, 1.32) | 1.22 | 1.19 (0.86, 1.65) |
| Average (ref) |  |  |  |  |
| Slower | 0.29 | 0.30 (0.11, 0.82) | 0.91 | 0.91 (0.63, 1.32) |
| Continuous model (Tanner stages/year) | 1.81 | 1.68 (0.83, 3.37) | 1.20 | 1.22 (0.85, 1.75) |

^a^Adjusted for maternal alcohol intake in pregnancy, maternal smoking in pregnancy, parental socioeconomic status in pregnancy, parental cohabitation in childhood, childhood internalizing and externalizing symptoms and behaviours, paternal and maternal smoking in childhood, paternal and maternal alcohol intake in childhood.

**Supplementary Table S11:** Boys' hash or pot use at age 18. Relative risk ratios for using hash or pot at age 18 compared to no use across pubertal developmental groups.

|  | Hash or pot, monthly | | Hash or pot, <1x/month | |
| --- | --- | --- | --- | --- |
|  | Crude | Adjusted (95% CI)^a^ | Crude | Adjusted (95% CI)^a^ |
| **Timing, genital** |  |  |  |  |
| Earlier | 1.36 | 1.24 (0.71, 2.16) | 1.26 | 1.27 (0.87, 1.86) |
| Average (ref) |  |  |  |  |
| Later | 1.01 | 1.08 (0.57, 2.07) | 0.67 | 0.65 (0.42, 1.00) |
| Continuous model (per year earlier timing) | 1.17 | 1.12 (0.93, 1.36) | 1.17 | 1.18 (1.05, 1.33) |
| **Timing, pubic hair** |  |  |  |  |
| Earlier | 0.74 | 0.68 (0.39, 1.19) | 0.88 | 0.86 (0.59, 1.24) |
| Average (ref) |  |  |  |  |
| Later | 0.54 | 0.53 (0.27, 1.06) | 0.66 | 0.60 (0.40, 0.91) |
| Continuous model (per year earlier timing) | 1.11 | 1.09 (0.88, 1.34) | 1.16 | 1.18 (1.02, 1.35) |
| **Timing, first ejaculation** |  |  |  |  |
| Earlier | 1.68 | 1.65 (0.93, 2.93) | 1.39 | 1.36 (0.95, 1.96) |
| Average (ref) |  |  |  |  |
| Later | 0.64 | 0.65 (0.32, 1.33) | 0.67 | 0.67 (0.42, 1.08) |
| Continuous model (per year earlier timing) | 1.40 | 1.40 (1.15, 1.71) | 1.28 | 1.27 (1.13, 1.42) |
| **Timing, voice break** |  |  |  |  |
| Earlier | 1.61 | 1.52 (0.84, 2.76) | 1.66 | 1.65 (1.11, 2.47) |
| Average (ref) |  |  |  |  |
| Later | 1.01 | 1.06 (0.51, 2.20) | 0.75 | 0.74 (0.46, 1.18) |
| Continuous model (per year earlier timing) | 1.15 | 1.13 (0.95, 1.35) | 1.22 | 1.23 (1.10, 1.37) |
| **Tempo, genital** |  |  |  |  |
| Faster | 0.61 | 0.58 (0.29, 1.16) | 0.76 | 0.78 (0.53, 1.14) |
| Average (ref) |  |  |  |  |
| Slower | 0.50 | 0.53 (0.26, 1.06) | 0.82 | 0.81 (0.55, 1.20) |
| Continuous model (Tanner stages/year) | 1.05 | 1.02 (0.58, 1.79) | 0.99 | 1.04 (0.72, 1.51) |
| **Tempo, pubic hair** |  |  |  |  |
| Faster | 1.12 | 1.19 (0.63, 2.26) | 0.71 | 0.74 (0.51, 1.08) |
| Average (ref) |  |  |  |  |
| Slower | 0.48 | 0.47 (0.25, 0.88) | 0.96 | 0.90 (0.63, 1.31) |
| Continuous model (Tanner stages/year) | 1.42 | 1.41 (0.90, 2.21) | 1.00 | 1.08 (0.82, 1.41) |

^a^Adjusted for maternal alcohol intake in pregnancy, maternal smoking in pregnancy, parental socioeconomic status in pregnancy, parental cohabitation in childhood, childhood internalizing and externalizing symptoms and behaviours, paternal and maternal smoking in childhood, paternal and maternal alcohol intake in childhood.
