# Supplementary material for: Timing and tempo of pubertal development and substance use in adolescence: a cohort study in the Danish National Birth Cohort
Source: Hum Reprod Open. 2025 Nov 18;2025(4):hoaf072. doi: 10.1093/hropen/hoaf072 (PMC12976676; doi:10.1093/hropen/hoaf072)
Supplement: hoaf072_Supplementary_Data [file hoaf072_Supplementary_Data.zip › Supplementary-figure-S1_post adjudication clean.docx]

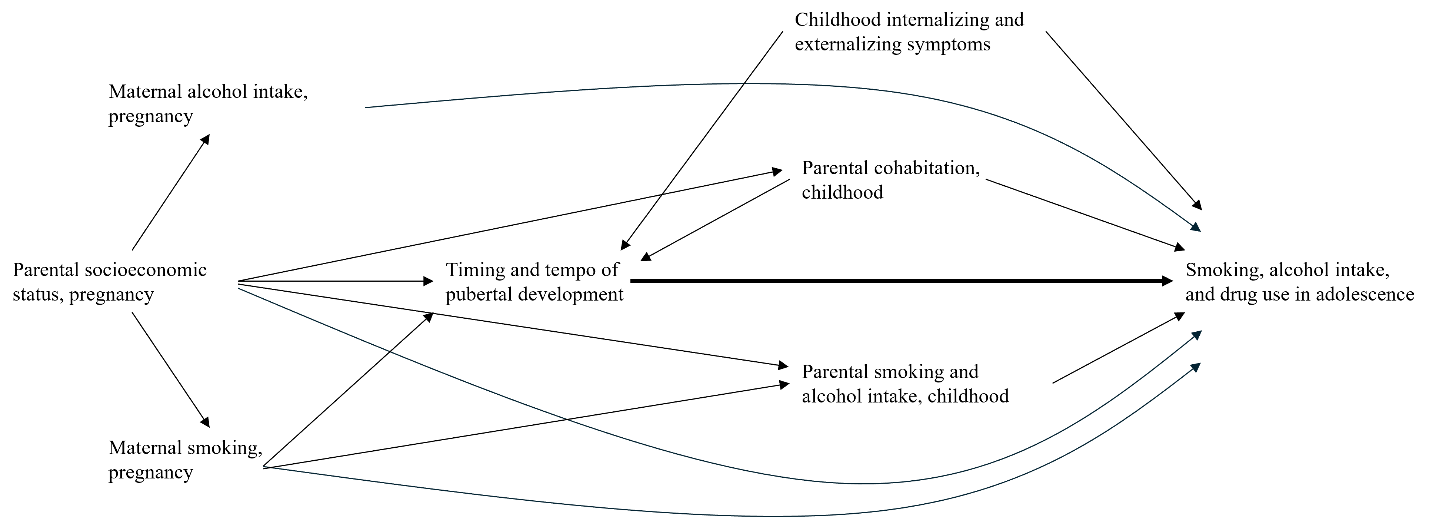


Supplementary Figure S1. Directed acyclic graph of the suggested causal structures of the association between timing and tempo of puberty and smoking, alcohol intake, and recreational drug use in adolescence.
